# Early response to nanoparticles in the *Arabidopsis* transcriptome compromises plant defence and root-hair development through salicylic acid signalling

**DOI:** 10.1186/s12864-015-1530-4

**Published:** 2015-04-24

**Authors:** Susana García-Sánchez, Irantzu Bernales, Susana Cristobal

**Affiliations:** Department of Physiology, Faculty of Medicine and Dentistry, University of the Basque Country UPV/EHU, Leioa, Spain; Gene Expression Unit, Genomics Facility of General Research Services (SGIker), Faculty of Science and Technology, University of the Basque Country UPV/EHU, Leioa, Spain; IKERBASQUE, Basque Country Foundation for Science. Department of Physiology, Faculty of Medicine and Dentistry, University of the Basque Country UPV/EHU, Leioa, Spain; Department of Clinical and Experimental Medicine, Health Science Faculty, Linköping University, Linköping, Sweden

**Keywords:** Nanoparticles, Nanotoxycology, *Arabidopsis*, Defence, Transcriptome, Stress, Systemic acquired response

## Abstract

**Background:**

The impact of nano-scaled materials on photosynthetic organisms needs to be evaluated. Plants represent the largest interface between the environment and biosphere, so understanding how nanoparticles affect them is especially relevant for environmental assessments. Nanotoxicology studies in plants allude to quantum size effects and other properties specific of the nano-stage to explain increased toxicity respect to bulk compounds. However, gene expression profiles after exposure to nanoparticles and other sources of environmental stress have not been compared and the impact on plant defence has not been analysed.

**Results:**

*Arabidopsis* plants were exposed to TiO_2_-nanoparticles, Ag-nanoparticles, and multi-walled carbon nanotubes as well as different sources of biotic (microbial pathogens) or abiotic (saline, drought, or wounding) stresses. Changes in gene expression profiles and plant phenotypic responses were evaluated. Transcriptome analysis shows similarity of expression patterns for all plants exposed to nanoparticles and a low impact on gene expression compared to other stress inducers. Nanoparticle exposure repressed transcriptional responses to microbial pathogens, resulting in increased bacterial colonization during an experimental infection. Inhibition of root hair development and transcriptional patterns characteristic of phosphate starvation response were also observed. The exogenous addition of salicylic acid prevented some nano-specific transcriptional and phenotypic effects, including the reduction in root hair formation and the colonization of distal leaves by bacteria.

**Conclusions:**

This study integrates the effect of nanoparticles on gene expression with plant responses to major sources of environmental stress and paves the way to remediate the impact of these potentially damaging compounds through hormonal priming.

**Electronic supplementary material:**

The online version of this article (doi:10.1186/s12864-015-1530-4) contains supplementary material, which is available to authorized users.

## Background

Nanoparticles (NPs) are materials with at least one dimension in the nanoscale (1–100 nm). The high surface-to-volume ratios and unique physicochemical properties of these materials have led to myriad practical applications, resulting in increased production and potential release into the environment. However, the effect of NP exposure on biological systems may differ from what is known about exposure to their bulk counterparts. Fate and transport studies have demonstrated that disposed NPs are present in nature at concentrations that might pose a risk [1,2].

Unicellular algae are widely used to test NP acute toxicity in the aquatic environment. At the cellular level, nano-forms of ZnO_2_, TiO_2_, or Ag can differently modify growth rates, cellular viability, and chlorophyll content compared to their corresponding bulk materials [3-5]. Subcellularly, alterations of organelles like chloroplasts and vacuoles and accumulation of NPs near the cell wall and plasma membrane have been observed [6,7]. At the molecular level, NPs increase reactive oxygen species (ROS) formation and lipid peroxidation to a higher extent that bulk materials [4,8-10]. However, many environmental pollutants can also induce ROS formation through an endogenous mechanism to alert for potential cell damage and increase environmental stress tolerance [11,12].

Physiological studies of higher plants have demonstrated that NPs up to 40 nm can be taken up by roots and travel symplastically through the vascular system, whereas larger NPs accumulate in the apoplastic space. Therefore, some of the damaging effects of NPs have been attributed to mechanical damage or clogging of plant structures, like plasmodesmata or stomata, which regulate water flux [7,9]. In maize TiO_2_- and clay-NPs reduced the size of cell-wall pores of roots and inhibited hydraulic conductivity and transpiration [13]. Consequently, plant molecular responses to mitigate NP-related damage might involve mechanisms to control water-stress, although this possibility has not been tested.

Research on crop plants has prompted further questions about the impacts of released NPs in agricultural production and the implications for the food chain [14]. Uptake, translocation, and generational transmission of carbonaceous nanomaterials have been demonstrated in rice [15]. Photothermal and photoacoustic methods have revealed the spatial distribution of carbon nanotubes (CNTs) in roots, leaves, and fruits of tomato plants [16]. In that work, the authors assayed the addition of four carbon-based materials to the plant growth medium and observed physiological responses for the single (SWCNTs) and multiwall (MWCNTs) CNTs only. MWCNTs over SWCNTs produced a maximum effect on biomass accumulation, thereof plants fed with MWCNTs were chosen for further examination by photothermal/photoacoustic scanning cytometry or microarray analysis. Imaging techniques detected MWCNTs in roots but also in the leaves and fruits of the plants exposed to NPs, and data were integrated in parallel with tomato microarray analysis to discover that MWCNTs induced changes in gene expression of stress- related genes. A number of differentially-expressed transcripts were identified that corresponded to 16 genes with known function, some of which are involved in plant responses to pathogen infection. This observation suggested that plants sense the penetration of nano-sized materials into their tissues as a biotic stress factor similar to pathogen or herbivore attack.

Gene expression analyses of the model plant *Arabidopsis thaliana* have provided new insights into the molecular mechanisms of plant response to NPs [17,18]. These two studies both reported gene expression changes upon long-term exposure to TiO_2_-NPs, ZnO-NPs, Ag-NPs, and fullerene of different sizes. There were important differences among NP doses, germination conditions, and plant developmental stages during exposure. Landa et al. [17] concluded that ZnO-NPs caused the most dramatic changes in gene expression, resulting in the up- and down-regulation of 660 and 826 genes, respectively, whereas TiO_2_-NPs only affected 80 and 74 genes, respectively, indicating minimal toxicity. ZnO-NPs and fullerenes up-regulated genes involved in functional responses to abiotic (salt or metal concentrations; water deprivation) and biotic stresses, whereas TiO_2_-NP exposure differentially regulated (both up and down) genes involved in both stresses, although data about the significance of these functional representation were not provided. In the study by Kaveh et al. [18], exposure to Ag-NPs was associated with the down regulation of genes involved in pathogen response, but a significant overlap was observed with the genes responding to bulk material. Importantly, some of the genes regulating hormonal stimuli and stress response were also identified as NP-responsive and connected with systemic acquired response (SAR), an enhanced immunity in tissues remote from the initial infection site [19,20]. SAR is triggered upon challenge by certain pathogens or, to some extent, by mechanical damage, *i.e.*, as a result of wounding by herbivore insects. SAR results in thickening of the cell wall and other physiological responses that enhance general plant defences in a non-specific way.

Here we produced a set of 16 comparable transcriptome profiles to monitor early changes in gene expression upon NP and stress exposure. We evaluated *A. thaliana* response to different types (metallic and carbonaceous) and sizes (4–80 nm) of NPs in comparison to biotic and abiotic stress inducers representing the most common environmental challenges for plants. Biotic stress was induced by infection with a necrotizing fungus (*Alternaria brassicicola*) or a hemibiotrophic bacterium (*Pseudomonas syringae* pv. tomato*, Pst*). These pathogens trigger plant defence and immune responses through mechanisms involving respectively, phytohormones like salicylic acid (SA), a key modulator of SAR, and jasmonic acid/ethylene pathways [21]. Abiotic stresses induced by hyper-saline conditions, drought, and mechanical wounding were also assayed. The effect of abscisic acid (ABA), the most studied stress-responsive phytohormone, which mediates stomatal closure and other responses to drought and osmotic stress [22], on the gene expressions and phenotypes of NP-exposed plants was also tested.

## Results

### Exposure of plants to pathogens, abiotic stress and NPs

Since the range of the transcriptional responses to stress depends on the growth conditions and developmental stage of the plant, synchronized hydroponic cultures were used for all exposures. Plantlets were grown in liquid MS medium until rosette leaves (8–12-leaf stage) emerged and begun aerial development. At this stage leaves can be inoculated with microbial pathogens as in the infection models used before to study immune response and roots are well developed to allow NP or salt uptake. Plants were infected with *A. brassicola* (**Abr**) or *P. syringae* (**Pst**) to study the transcriptional response to biotic stress in 2 of the 16 conditions that were assayed. Infected plants developed chlorotic leaves or other macroscopic signals of a plant hyper-sensitive immune response by the second day post infection (dpi), indicating that mechanisms of defence against biotic stress were already activated. Similarly, physiological responses to saline stress were observed 2 days upon addition of 100 mM NaCl (**NaC**) to the medium. Drought (**Drou**) and wounding (**Wou**) stress were induced as described in the methods section to assay a total of 3 conditions for abiotic stress that have been connected before with NP-induced damage.

NPs were added to the cultures at the same time that the other stressors and incubated with the plants for 2 days before collection. We performed preliminary dose- and size-dependent measurements of the effects of NPs on plant biomass under these particular growth conditions. These experiments narrowed the range of concentrations at which NPs had measurable effects on plant growth to 0.2–25 μg/mL, similar to previous studies [7,16,23]. We assayed NPs made of 3 different materials, Ag, TiO_2_ and carbonaceous materials (COOH-functionalised MWCNTs, COOH-MWCNTs), and diameters ranging from 10 to 80 nm for Ag-NPs, 10 to 40 nm for TiO_2_-NPs and 4–12 nm for COOH-MWCNTs. This made a total of 8 different conditions (**AgNP10**, **AgNP20**, **AgNP40**, **AgNP80**, **TiO**_**2**_**NP10**, **TiO**_**2**_**NP20, TiO**_**2**_**NP40** and **COOH-MWCNT**) for NP-exposed plants. Bulk materials that release equivalent metallic ions (**AgNO**_**3**_ and **TiO**_**2**_) were added to 2 additional series of plants and one more condition was assayed in which plants were supplemented with ABA 12 h. before addition of COOH-MWCNTs (**COOH-MWCNT+**). Combined exposure to NPs and ABA was performed to determine whether NP-induced effects at the transcriptomic level could be prevented or reverted with ABA treatment. This hormone activates physiological responses to drought, salt, and other abiotic stresses through a complex network of transcriptional regulators. Exogenous addition of ABA can mimic the endogenous accumulation of the hormone that triggers stress responses in the tissues and increases plant tolerance to saline stress [24-26].

### Transcriptome analyses of exposed plants

We examined the changes that occurred in the transcription of 26,184 genes represented in the At V4 microarray. Hybridization signals from the cDNA of plants exposed to the 16 conditions described above were normalised relative to the values of non-exposed plants (Additional file 1). Changes in these normalised gene expression values were further analysed.

**Figure 1:**
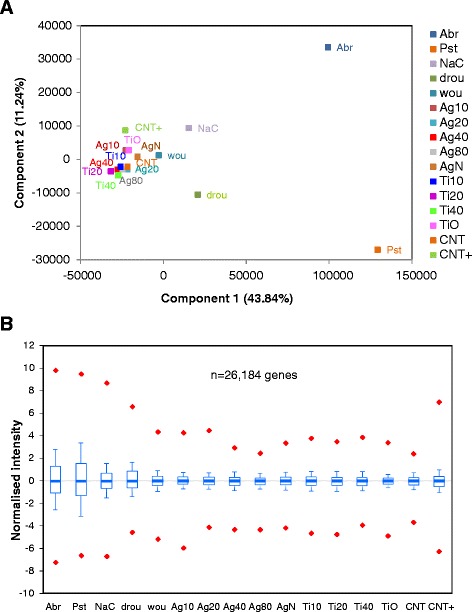
Principal Component Analysis and distribution of normalised expression ratios for the total of genes analyzed. **(A)** PCA was performed to reduce the dimensions of expression data to 4 components. Components 1 to 4 explained respectively 43.84%, 11.24%, 10.58% and 8.54% of variation in the data. The first and second components were used as the X and Y axes to plot values from the 16 conditions in this study. Ag10, Ag20, Ag40, Ag80 and AgN represent the addition of Ag-NPs of 10-, 20-, 40- and 80-nm diameter or AgNO_3_ bulk material, respectively. Ti10, Ti20, Ti40 and TiO the addition of TiO_2_-NPs of 10-, 20-, 40-nm diameter and TiO_2_ bulk material. CNT and CNT+ indicate COOH-MWCNTs added to non-supplemented or ABA-supplemented plants. **(B)** Box plot of the expression ratios for the 26,184 genes analysed with the AtV4 microarrays. Boxes represent 10th and 90th percentiles, with the median (Q_2_) in the middle line of the box. Minimum and maximum values (snapped to Q_1_-1.5 x IQR and Q_3_ + 1.5 x IQR, IQR = Interquartile Range) are shown by the end of the vertical lines (whiskers) and upper or lower outliers by red diamonds.

Principal Component Analysis (PCA) was performed to detect major trends in the expression data. The first and second components of the analysis, which captured respectively 43.84% and 11.24% of the variation, were plotted to visualize similarities among samples (Figure 1A). The scatter-plot shows that all NP exposures clustered together, independently of material (**TiO**_**2**_, Ag or carbonaceous) or size in the nano scale. The biotic stresses induced upon pathogen challenge (samples **Pst** and **Abr**) were discriminated from other conditions to the right side of the plot. The abiotic stressors (**NaC** and **Drou** and, to a higher extent, **Wou**) graphed closer to the NP exposure samples. Thus, plants exposed to NPs show similar transcriptional behaviours, and pathogen challenge, saline or drought stress -in this order- cause major changes in the transcriptome when compared to NP exposure. In support of this, box-plot representation of normalised expression ratios shows that the ranges of expression changes under these stress conditions are spanned (Figure 1B). For example, 90% of the genes in the *Pst*-infected plants had expression ratios between −1.3 and 1.5, whereas this range was only −0.4 to 0.3 in samples exposed to NPs. Pre-treatment with ABA in COOH-MWCNT-exposed plants did not decrease the range of expression changes to that of non-exposed plants, suggesting that the addition of this hormone had side effects on the plant transcriptome.

### Identification and functional classification of genes with significant transcriptional responses to NPs

**Figure 2:**
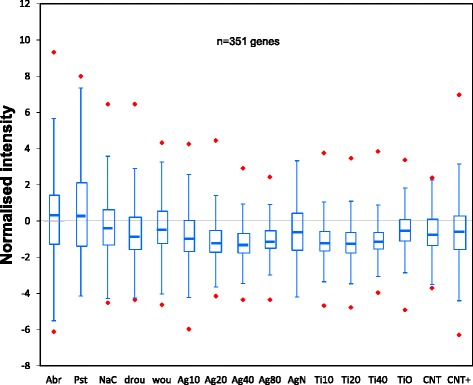
Quartile distribution of the expression ratios for the 351 NP-responsive genes. Boxes represent Q_1_ and Q_3_ quartiles and the middle line of the box is the median (Q2). Whiskers show the minimum and maximum values (snapped to Q_1_-1.5 x IQR and Q_3_ + 1.5 x IQR) and red diamonds outliers.

Microarray data was statistically analysed to identify differentially-expressed genes that responded to all NP treatments, irrespectively of particle size or type. The analysis recovered a set of 351 genes with significant (*p* < 0.05) changes in expression (Additional file 2). Quartile (Q) representation of the expression changes for this set (Figure 2) showed that most were repressed (Q_3_ < 0) in NP-treated samples, and median expression ratios were low (Q_2_ < −1) compared with those of non-exposed plants.

**Figure 3:**
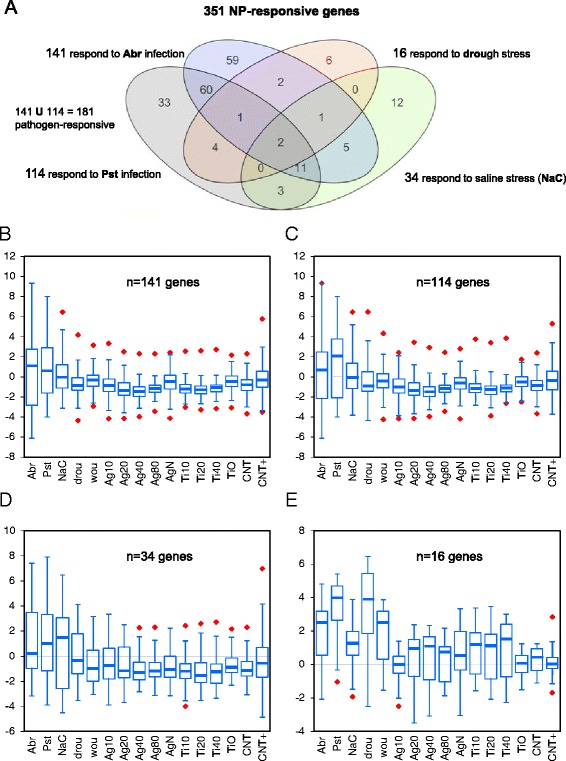
Distribution of the expression ratios for the 4 subsets of stress-responsive genes within the 351-gene set. **(A)** Venn diagrams represent the 4 subsets of 141 (**Abr**), 116 (**Pst**), 34 (**NaC**) and 16 (**Drou**) stress-responsive genes that were included in the group of 351 NP-responsive genes, with the number of overlapping genes in each intersection. **(B, C, D** **and** **E)** Distribution of the expression ratios within each subset. Boxes and middle line represent Q_1_-Q_3_ quartiles and the median of the distribution. Whiskers show the minimum and maximum values (snapped to Q_1_-1.5xIQR and Q_3_ + 1.5xIQR) whereas red diamonds show outliers.

To detect genes that were empirically (rather than *in silico*) responding to biotic or abiotic stress, we performed independent analyses of **Abr**, **Pst**, **NaC**, **Drou**, and **Wou** samples to define five sets of biotic- or abiotic-stress-responsive genes under our experimental conditions. These sets were overlapped with the group of 351 NP-responsive genes to produce four subsets of 141 (**Abr**), 114 (**Pst**), 34 (**NaC**), and 16 (**Drou**) differentially-expressed genes, with broad overlap between the **Abr**- and **Pst**-responsive subsets (Figure 3 and Additional file 3). Thus, most of the 351 genes with significant responses to NPs were also regulated upon pathogen challenge in our hydroponic growth model. In contrast, none of the significant wounding-responsive genes were included in the 351-gene set.

**Figure 4:**
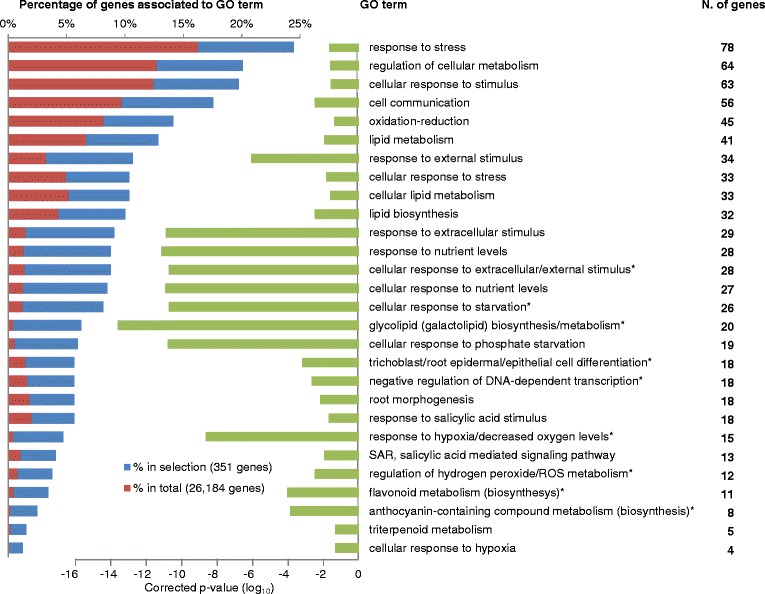
GO enrichment analysis of the 351-gene set. The percentages of genes that are associated to a GO-term are shown in the left bars for the set of 351 differentially expressed genes and for the total set of 26,184 genes Enrichment scores (*P*-values) are represented by the bar graph in the right. The number of genes associated to each GO term is shown in the right column. Only GO-terms in the category of Biological Process are shown. Some descendant terms (*) that contain the same genes have been merged and represented as a single category bar for simplicity (detailed values in Additional file 4).

Functional *in silico* classification of the 351 genes was achieved via Gene Ontology (GO) analysis. A total of 60 GO terms were significantly (*p* < 0.05) enriched in the three supercategories of Biological Process, Cellular Component, and Molecular Function (Additional file 4). GO terms in Biological Process are shown in Figure 4. The most represented term in the 351-gene set is response to stress, which included 78 genes from the set (24.5%) and was significantly (*p* = 9.4 × 10^−5^) enriched with respect to the total of 26,184 genes (16.2%). Additional analyses of some of these functional classes are presented in the following sections. In Cellular Component, a significant percentage of the 351 genes corresponded to extracellular proteins (66 genes, *p* = 3.7 × 10^−9^), whereas none of the other sub-cellular compartments were over-represented (Additional file 4). The most enriched category within Molecular Function was alkaline phosphatases.

### Transcriptional responses to NPs include repression of pathogen-activated genes and salicylic acid-mediated pathways

**Figure 5:**
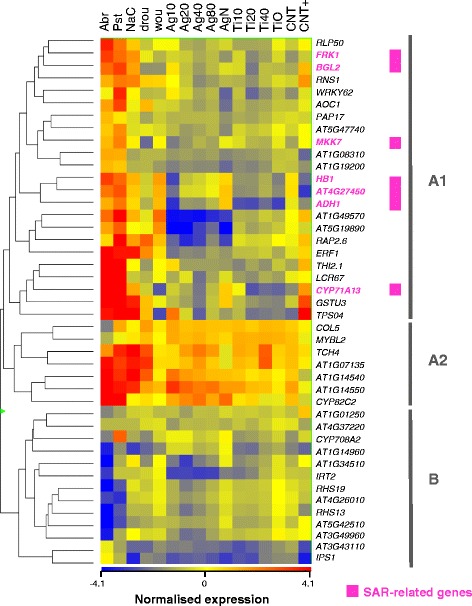
Clustering of the stress-annotated, pathogen-responsive genes in the 351-gene-set. Hierarchical clustering was represented for the 43 gene-entities using Euclidean distances and following Ward’s Linkage rule. Branch A_1_ includes genes that are activated upon pathogen challenge and repressed in NP-exposed plants. This branch contains most SAR-responsive genes. Branch A_2_ corresponds to genes activated by pathogen challenge and also, but to a lesser extent, by NP exposure. Branch B is formed by the genes repressed both by pathogen and NP-exposure (box-plot representations for each branch in Additional file 5).

**Abr**- and **Pst**-responsive genes in the 141- and 114-gene subset were generally repressed in all NP treatments (Figures 3B and C). We focused on genes previously associated with stress responses by the GO annotation and for which more information was available. Hierarchical clustering grouped them into two main branches (Figure 5) which roughly corresponded to genes that were up- (branch A) or down-regulated (branch B) by one or both biotic stresses. Most of the genes in both branches (branches A1 and B) were repressed in all NP-treated samples (box-plot representation in Additional file 5) and only 7 genes (branch A2) responded to NP treatments with log ratios above 0, although overall these genes were up-regulated more by pathogen challenge than by NP exposure.

Most pathogen-responsive genes that were activated during our **Abr**/**Pst** challenges are involved in early defence signalling and represent main effectors of the plant immune response that triggers SAR upon pathogen infection. Such was the case with *FRK1* [AT2G19190], an inducible kinase in the pathway that activates the basal immune response upon perception bacterial flagellin [27], and other genes in the enriched GO category of SAR via SA signalling (Figure 4). These genes were all significantly repressed by NP exposure (detailed clustering in Additional file 6). These data suggest that repression of pathogen-induced genes and SA response is a common feature of NP exposure.

### Exposure to NPs represses expression of phosphate-starvation and root-development genes

**Figure 6:**
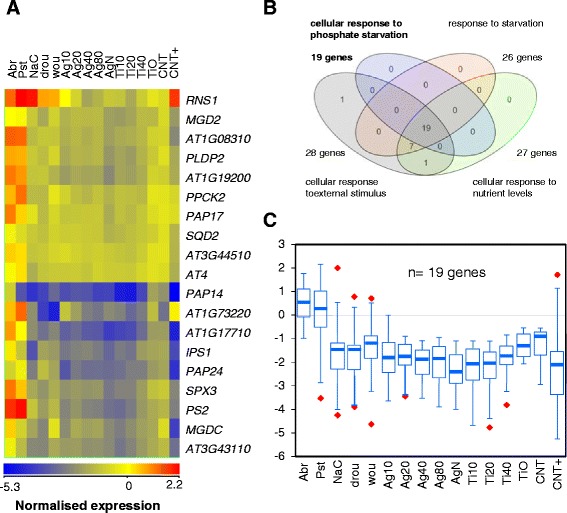
Repression of the phosphate-starvation genes upon NP exposure and overlapping GO categories. **(A)** The 19 NP-responsive genes that are included in the enriched GO category of phosphate starvation response are shown clustered. **(B)** Overlapping of the enriched categories that contain most phosphate-starvation genes **(C)** Box-plot representation of Q_1_, Q_3_ and median expression ratios of the 19 phosphate-starvation genes; whiskers show minimum and maximum values and red diamonds outliers.

The most regulated genes (with the greatest fold-changes in expression compared with unexposed plants) in the set of 351 differentially-expressed genes were related to the phosphate starvation response (Figure 6). The enriched GO subset included 19 genes that are also present in the more general GO categories of cellular response to starvation, cellular response to nutrient levels, and cellular response to extracellular stimulus. Purple-acid phosphatases (*PAP14* [AT2G46880], *PAP17* [AT3G17790], and *PAP24* [AT4G24890]) and other genes in the set that are induced by phosphate starvation (*IPS* or *PS*) were strongly repressed in all NP-treated samples, as confirmed by RT-qPCR (Additional file 7).

**Figure 7:**
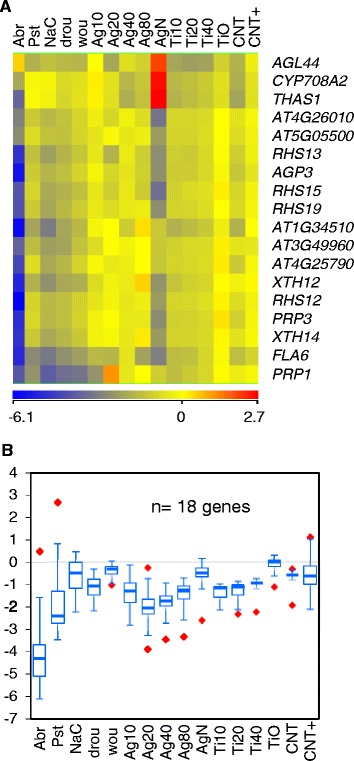
Repression of the root morphogenesis genes upon NP-exposure. **(A)** The 18 NP-responsive genes included in the enriched GO categories of trichoblast differentiation/root morphogenesis are shown. **(B)** box-plot representation of the Q_1_, Q_3_ and median expression values for the set.

Transcriptional responses to phosphate starvation have been widely studied [28-30] and result in important changes to root morphology by promoting the inhibition of primary root growth and the formation of lateral roots, as well as the proliferation of root hairs [31,32]. All these changes increase the capacity of plants to absorb available phosphate from the soil and constitute a local response to external phosphate starvation [33]. Sensing of low phosphate availability also triggers long distance signals to systemic tissues that activate phosphate homeostasis mechanisms via increased phosphate transport, recovery, and recycling by modulating the expressions of high-affinity transporters, secreted phosphatases, and phospholipid catabolism enzymes. In NP-exposed plants, we observed repression of phosphate homeostatic enzymes as well as genes directly involved in root hair development from epidermal trichoblasts. Root-hair-specific genes (*RHS12* [AT3G10710], *RHS13* [AT4G02270], *RHS15* [AT4G25220], and *RHS19* [AT5G67400]) and 14 other genes with roles in root hair emergence and differentiation [34-36] constituted the enriched subsets of trichoblast/epidermal cell differentiation and root morphogenesis genes in the 351-gene set (Figure 7). Unlike the phosphate-starvation genes, root hair genes were repressed, rather than activated, during **Abr**/**Pst** stress (Figures 6C and 7B). Because many of them encode cell-wall modification enzymes that shape the trichoblast, differential expression of this group likely involves structural changes in the roots of plants exposed to NPs, which is not necessary implicit in the transcriptional regulation of phosphate homeostatic enzymes.

### Exposure to NPs increases bacterial survival in local and distal leaves of plants infected with *Pst*

We next tested some predictions of the transcriptional reprogramming observed upon NP exposure on the phenotypic response of the plant to biotic stress. A significant number of NP-repressed genes are activated upon pathogen infection and are involved in SAR, therefore exposure to NPs before pathogen challenge might compromise a plant’s ability to respond to infection. However, if this effect were modulated by SA, exogenous supplementation with the hormone might compensate for it and prevent the NP-induced phenotype.

**Figure 8:**
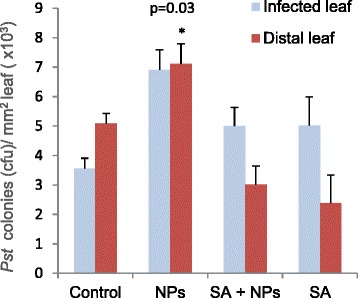
Bacterial growth upon *Pst* infection of NP-exposed and non-exposed plants. Mayor rossete leaves of plants were inoculated with a suspension containing 10^4^ colony forming units (cfu) of the *Pst* strain or with a mock solution (control) 24 h after exposure to NPs.cfu were counted in inoculated and distal leaves 2 days after inoculation. The effect of supplementation with SA 12 h before NP addition was measured in another series of plants. Bacterial growth in distal leaves of NP-treated plants was significantly increased respect to the control plants.

We measured the spread of the hemibiotrophic pathogen *Pst* in the leaves of NP-exposed plants. Rosette leaves were inoculated with the bacterium 1 day after addition of NPs to the MS growth medium and compared with NP-unexposed control plants. Another series of plants were treated with SA before the addition of NPs. Bacterial growth in local (inoculated) and distal (systemic) leaves was determined 2 days after inoculation (Figure 8). The NP exposure was associated with increased bacterial growth, both in local and distal leaves of infected plants, compared with the control. Control plants experienced only about half the level of infection in NP-exposed plants, consistent with an intact (non-NP-repressed) transcriptional mechanism that can activate pathogen-resistance genes. Exogenous supplementation of SA prevented the bacterial growth increases associated with NP treatment in the distal leaves, where systemic signalling by SA enhances resistance to infection.

### Exposure to NPs inhibits root hair development

**Figure 9:**
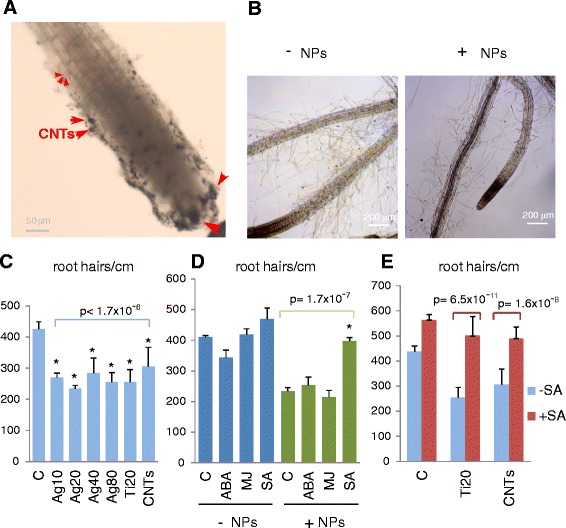
Inhibition of root hair production upon NP exposure and effect of SA pre-treatment. **(A)** Accumulation of NPs in the root cap. Microscope photographs of the roots of plants exposed to COOH-MWCNTs 4 days upon addition of the NPs to the growth medium: black aggregates appear around the root cap. **(B)** Microphotographs of the lateral roots of plants exposed to Ag-NPs (20 nm); NP-exposed roots show a “hairless” phenotype. **(C)**. Number of root hairs per cm of root in plants exposed to Ag-NPs, TiO_2_-NPs, and COOH-MWCNTs, compared to control (non NP-exposed) plants. A significant decrease is observed in all the plants treated with NPs. **(D)** Effect of the pre-treatment with ABA, Methyl Jasmonate (MJ) and SA phytohormones before exposure to 20 nm Ag-NPs; only the addition of SA can prevent the decrease in the number of root hairs/cm observed upon exposure to NPs. **(E)** Pre-treatment with SA prevents the reduction in root hair density upon treatment with TiO_2_-NPs and COOH-MWCNTs.

Transcriptional repression of the root development genes predicted an altered root phenotype in plants exposed to NPs. Absorption of NPs in the root cap and accumulation in the columella has been shown in A. *thaliana* plants that were germinated and fed with high concentrations of 40 nm AgNPs [7]. Similarly, we observed the accumulation of COOH-MWCNTs around the root caps of plants growing under our hydroponic conditions (Figure 9A). Shortening of the root tips and other alterations to root morphology have been described in *A. thaliana* and other plants exposed to some types of NPs [23,37]. However our transcriptome analysis pointed to a specific effect of NPs on the epidermal cells that originate root hairs and indicated that *RHS* genes were repressed by all types of NPs tested in our experiments. Root hairs substantially increase the root surface area in contact with the soil, and most of the water and nutrients that enter the plant are absorbed through them. Thus, their development is significantly affected by environment stimuli and stress signals [38,39].

We performed detailed microscopic observations to quantify the effects of NPs on root morphology under our plant growth conditions. Superficially, NP-treated roots resembled some “hair-less” mutants [34,40] (Figure 9B). Quantification confirmed that NP-exposure significantly decreased root hair density regardless of the size or type of NP (Figure 9C). Because hormonal balance regulates root development, and ABA has been shown to be locally involved in the readjustment of root morphology under abiotic stress conditions [41,42], we tested the effects of exogenous supplementation with different hormones (SA, ABA, and methyl jasmonate (MJ)) before the addition of NPs. Supplementation with SA prevented the reduction in root hair density caused by NP exposure (Figure 9D). Further assays with TiO_2_ NPs and COOH-MWCNTs confirmed that supplementation with SA could rescue the phenotypic effects of all types of NPs on root hair development (Figure 9E).

## Discussion

Nanoparticle exposure had less of an impact on the plant transcriptome than the other stress conditions assayed in this work (Figure 1). As measured by genome expression changes, biotic stress represented the major challenge for these plants, whereas saline or drought stresses have lesser effects, although still greater than that of NP exposure. The plant–pathogen system or abiotic stresses used here may represent harsher environmental conditions than NP exposure. However none of the stresses assayed here posed severe challenges to plant survival, because our seedlings (including the NP-exposed plants) completed their vegetative life cycles, bolted, and eventually flowered 1–2 weeks after exposure. Exposure to NPs under these conditions induced significant changes in gene expression as well as measurable phenotypic differences (Figures 2, 4, and 9), so their effects at these doses cannot be considered negligible. Therefore, compared with other environmental challenges, *A. thaliana* has the genetic resources to withstand NP-induced stress, although to a great extent the response will depend on the concentrations and activities of the nanomaterials in the plant environment [1]. More interestingly, in our analysis, all tested types and sizes of nanoparticles plotted in the same area in the PCA, supporting the idea of common regulatory mechanisms in the *Arabidopsis* response to NPs that could be exploited to prevent negative effects on plant development.

Down-regulation of gene expression was an overall response to NPs. The Q_3_ values for the 351 differentially-expressed genes were below zero in all the NP-treated samples (Figure 2). Overall, this down-regulation affected all the gene subsets in the GO categories represented in the Figure 4, except for cellular response to hypoxia (Additional file 8). The subsets of 141, 114, and 34 genes that responded to biotic or saline stress were also down-regulated in the NP samples (Figure 3B, C, and D), and only the 16 drought-responsive genes responded to NPs with increased expressions.

Transcriptional repression is suspected to be an important mechanism to keep stress responses under tight control [43-45]; early response to NPs might involve preventive repression of gene expression to allow genetic checkpoints to be readjusted before further responses are triggered. Alternatively, other mechanisms different from transcriptional repression could be mediating the down-regulation of NP-responsive genes. Interestingly, TiO_2_-NPs induced the expression of microRNAs in tobacco [23]; these molecules are important repressors of mRNA processing that function in plant responses to environmental stress [46,47].

A significant number of NP-responsive genes were involved in the biotic stress response in our study, in agreement with data from other authors that identified differentially-expressed genes upon long-term exposure to some types of NPs [17,18]. We assayed NPs of different materials and sizes and look for common patterns in the transcriptional response to NP exposure. NPs induced early differential expression of 181 genes that, under similar growth and developmental conditions, were also regulated by pathogen challenge (Figure 3), and we show that the genes up-regulated by pathogens were down-regulated by NPs (Figure 5). In particular, down-regulation affected a significant proportion of genes that are key components of the pathogen-detection pathways that activate SAR and SA signalling, like *FRK1* (Figure 5 and Additional file 6). The repression of the SAR response that we observed 2 days upon NP exposure has been corroborated in experiments with *Arabidopsis* exposed for 10 days to Ag-NPs [18]. The long-term repression of these genes would have important consequences for the plant’s capacity to withstand biotic stress under environmental conditions. In a phenotypic analysis, we showed that exposure to NPs and subsequent infection with phytopathogenic bacteria resulted in increased bacterial colonization of the plant that could be prevented by exogenous application of SA (Figure 8). In agreement, exogenous SA can prevent transcriptional repression of *FRK1* by NPs and up-regulates this gene to the levels of infected plants (Additional file 7).

The GO classification showed that phosphate-starvation genes were significantly over-represented (*P* = 1.3 × 10^11^) in the set of NP-responsive genes (Figures 4 and 6). Genes in this category were the most strongly repressed by treatment with all types of NPs and included genes encoding enzymes like *PAP* phosphatases, as well as non-protein-coding genes such as *IPS1* [AT3G09922], that is involved in the complex regulation of phosphate-starvation responses through microRNA production [32,48]. Consistent with this finding, other non-overlapping genes in the GO categories of NP-regulated genes were physiologically connected to the phosphate-starvation response. This was the case not only for the genes related to root hair development but also the over-represented categories of anthocyanin and flavonoid biosynthesis (Additional file 4). Anthocyanins accumulate in the roots of phosphate-starved plants and are often used as metabolic markers of phosphate starvation, whereas low nitrogen induces roots to accumulate their precursors, flavonoids [29,49].

Phosphate starvation and other stress signals shape the plant root and can induce root hairs to proliferate to increase nutrient and water absorption. Root hairs are formed by the differentiation of old epidermal cells that originate from the apical meristematic region. These epidermal cells become either root hair cells or non-hair cells depending on whether they are located over the intercellular space between two underlying cortical cells or over a single cell. Transcriptomic studies have revealed the temporal patterns of gene expression during the development of hair and not-hair cell lines and dissected the complex regulatory network involved in epidermis cell differentiation [34]. These studies define a group of 154 hair-cell genes and 54 non-hair-cell genes within the “core” of 208 root epidermal genes that were regulated in opposite directions in “hairy” and “hair-less” phenotypic mutants. We observed consistent, NP-induced, down-regulation of 18 of the 19 “core” root epidermal genes in the group of 351 differentially-expressed genes, and all were hair-cell genes. Moreover, Q_3_ values in the box-plot representations of the 155 hair and 54 non-hair genes show that down-regulation was specific of hair-cell genes (Additional file 9). Our phenotypic observations demonstrated a significant decrease in root hair densities with all the NP types assayed (Figure 9C). Other works have shown that exposure to NPs affects root development, although the transcriptional mechanisms involved were not elucidated. Ultrasmall TiO_2_-NPs accumulate in the roots, but not shoots, of *Arabidopsis* seedlings [50] and cause isotropic growth of root epidermal cells and swelling of root tips as soon as 36 h after NP addition [51]. This effect has been attributed to the disruption of microtubular networks by NPs, thus increasing the 26S proteasome workload to degrade the depolymerised tubulin. However, our transcriptome analysis did not suggest proteasome alterations.

Similar to the increased sensibility of leaves to *Pst* infection, the hairless-like root phenotype induced by NPs could be prevented by supplementation with SA but not other hormones like ABA or MJ (Figure 9D). Pre-treatment or “priming” of plants with hormones may improve resistance to future exposure to environmental stress [52,53] and, under our conditions of NP-induced stress, SA seemed to have a priming effect to reduce pathogen proliferation and the root hair-less phenotype.

Genes up-regulated by NP exposure are included in the GO category of cellular response to hypoxia and in the subset of drought-responsive genes (clustered in Additional file 10). The first group includes two peroxidase superfamily proteins expressed in root tissues ([AT1G14550] and [AT1G4540]) and *CYP82C2* [AT4G31970], which modulates jasmonate-induced root growth inhibition and defence gene expression [54]. Early induction of peroxidase and superoxide-dismutase genes has been reported both in plant protoplasts and in leaves upon injection of COOH-MWCNTs [55] and was connected to programmed cell death of protoplasts. Drought stress in our study affected a number of NP-induced genes with previously unknown functions as well as the better characterized *MYBL2* [AT1G71030] and *COL5* [AT5G57660] transcription factors. *MYBL2* is a proto-oncogene homologue involved in *BES1* co-repression in the brassinosteroid signalling pathway [56] and was more recently identified as a dehydration stress memory gene regulated by multiple signalling pathways, including the ABA, jasmonic acid, and SA pathways [57]. *COL5* is a member of the CONSTANS-LIKE family of flowering regulators involved in the response to water deprivation [58]. Interestingly, pre-treatment with ABA, as well as with MJ or SA, can decrease the transcriptional induction of these genes by COOH-MWCNTs, and in particular, SA can by-pass NP-induced activation and regulate their expressions down to the levels of ***Abr*** infection (Additional file 7). *MYBL2* and *COL5* also respond to **Abr**/**Pst** pathogen challenge and, as members of very complex families of transcription factors, might play important roles in integrating hormonal signalling in the plant response to NP-induced stress.

## Conclusions

We evaluated the transcriptome changes of *Arabidopsis* after brief exposure to NPs and compared them with those under biotic or abiotic stresses that represent common environmental challenges for plants. Principal Component Analysis clusters together transcriptomes of plants exposed to NPs of different materials and sizes in the nano-scale. The study of transcriptomic patterns could distinguish the impacts of all tested NPs from those of other stressors and defined a set of NP-responsive, differentially expressed genes. NP exposure repressed a significant number of genes involved in the response to pathogen challenge and increased bacterial survival during an experimental infection with ***Pst***. The mechanism involved in SAR via SA signalling could by-pass NP-induced repression and reduce bacterial colonization. In addition, NP-induced repression largely involved genes activated by phosphate-starvation and other conditions that promote root hair development, but supplementation with SA could remediate the resulting root hair-less phenotype. Overall, the basic molecular mechanisms of the early response of *Arabidopsis* to NPs were based on transcriptional repression and had a common pattern regardless of the composition of the different NPs.

## Methods

### Plant growth conditions

*Arabidopsis thaliana* accession Columbia-0 (Col-0) was the genetic background used in this study. Seeds were surface-sterilized in a 20% bleach/0.05% Tween-20 solution, rinsed five times in sterile deionised water, and then sown on Petri dishes containing ½ Murashige and Skoog medium (½ MS basal salts, 2% glucose, 0.6% agar, pH 5.7). Plates were sealed and stored for 3 days at 4°C in darkness before incubation in a controlled environment growth chamber at 22°C, 70% relative humidity, and 200 μM × m^2^/s of cool white fluorescence illumination (12 h light/12 h dark). Seven-to-ten-day-old seedlings with at least 1-cm-long roots were selected and individually transferred to test tubes containing 5 mL of liquid ½ MS and incubated for 1–2 additional weeks with continuous shaking at 50 rpm. Plants were incubated until roots had grown to fit within the liquid volume of the culture and plants stop floating (about 3 weeks after seeding). Plants with developed aerial rosettes (8–12-leaf stage) were then selected for further treatment with NPs, microbial pathogens, or abiotic stress. For each biological replicate, eight plants were individually grown in test tubes and treated with NPs or other stressors before RNA extraction. Unexposed plants used as the controls were treated similarly except for the lack of NPs in the media.

### Treatment of plants with nanoparticles

All NPs used in this study were obtained from commercial manufacturers who performed detailed characterisation of the diameter, mass concentration, spectral properties, and Z-potential. Ag-NPs were provided as a 0.02-mg/mL suspension in aqueous citrate buffer by either Ted Pella Inc. (20, 40 and 80 nm; Redding, CA, USA) or Sigma–Aldrich (10 nm; St. Louis, MO, USA). TiO_2_ anastase-NPs (10, 20, and 40 nm) were purchased from Nanograde LLC (Stäfa, Switzerland). COOH-functionalized, multi-walled PELCO® Carbon Nanotubes of 4–12 nm diameter and 5–15 μm length were obtained from Ted Pella Inc. The introduction of carboxylic functional groups onto the surface of these hydrophobic nanostructures enhances their water dispersion and creates more homogenous solutions, and it has been shown by other authors that carbonaceous NPs solubilised in this way can be up taken from MS medium and transported to the areal tissues of the plant [16]. PELCO® COOH-MWCNTs were added to sterile water to make a 1.25 mg/mL stock solution and vortexed for 1 min. immediately before their addition to the MS medium. NPs were added to MS solutions of 3-week-old plants at the end of a light period and to a final concentration of 0.2 μg/mL for Ag-NPs, 20 μg/mL for TiO_2_-NPs, or 25 μg/mL for COOH-MWCNTs. Plants were grown for two additional days in the presence of NPs in climate chamber and with continuous shaking, then collected for RNA extraction. Under these conditions, plants that were not collected for RNA extraction and remained in the climate chamber were able to complete their vegetative cycles and initiate bolting within 2 weeks of exposure, although the timing was variable. Fresh weights of plants were determined 7 days after NP addition and average were lower than those of the unexposed control plants, but never less than 84% of the control weight. Bulk materials (AgNO_3_ or anastase TiO_2_) were provided by Sigma–Aldrich and added to the same concentration as the corresponding NPs.

### Infection with microbial phytopathogens

*Alternaria brassicicola* DSM-62008 strain was purchased from the DSMZ-German Collection of Microorganisms (Braunschweig, Germany) and cultivated following their recommendations. Conidia from freshly-grown cultures on agar plates were collected and resuspended in sterile milli-Q water (Millipore, Billerica, MA, USA) to 10^5^ spores/mL. Five microliters of this suspension were used to inoculate the largest leaf of 3-week-old plants; leaves were collected 2–3 days after infection, depending on macroscopic symptoms of disease. Symptoms mostly consisted of yellowing around the inoculation area and occasional black spots. Under the microscope, dead cells appeared at the borders of the wound, with fungal mycelia invading the surrounding areas.

*Pseudomonas syringae* pathovar *tomato* DC3000 strain (wild-type, Rif^r^) [59] was a generous gift from Dr Jens Boch (Martin Luther Universitat, Halle, Germany) and was cultured and inoculated into plant leaves as previously performed in a plant-pathosystem model to study immune response [60]. Collection of plants for RNA extraction was as described for *A. brassicicola*.

### Abiotic stress conditions and hormone treatments

Exposure to abiotic stress was performed according to previous works on the transcriptional regulation of typical stress-responsive genes [61,62]. Hypersaline conditions were reproduced by adding 100 μL of a 5-M sterile NaCl solution to the 5 mL of liquid MS medium. For drought-stressed plants were removed from their test tubes and exposed to air on a 3MM Whatman paper (GE Healthcare, Little Chalfont, UK) for 2.5 h. Mechanical wounding was produced by crushing rosette leaves several times with plastic forceps [63]. Except for the drought-stress condition, all plants were collected 2 days post-treatment for RNA extraction. Hormone treatments were performed 12 h before the addition of NPs on 3-week-old plants grown as described above. ABA was added to the 5-mL culture to a final concentration of 3 μM from a freshly-prepared, 3-mM stock solution [64]. SA was added to 0.1 mM from a solution containing 10 mM SA and 0.01% Silwet L-77 [65]. MJ treatment involved dropping 0.4 μL of a 0.5% solution in ethanol onto the cellulose cap of the test tube containing the plant; test tubes were sealed with Parafilm (Bemis, Neenah, WI, USA) and introduced into an hermetic 20-L container that was shaken in the climate chamber. Control plants were treated similarly, except for the absence of phytohormones in their solutions.

### Microarray hybridisation and transcriptomic analysis

To allow significant transcriptome measurements, all treatments were performed in quadruplicate for each series of eight plantlets. Plants were collected from the test tubes and put on 3MM Whatman paper to remove excess MS and then frozen in liquid nitrogen prior to homogenization, as described before [60]. Total RNA was extracted with TriPure Isolation Reagent (Roche, Penzberg, Germany) and quantified using a NanoDrop ND-1000 UV–VIS spectrophotometer (Wilmington, DE, USA). RNA quality and integrity were assayed by Lab-chip technology on an Agilent 2100 Bioanalyzer with Agilent RNA 6000 Nano Chips (Santa Clara, CA, USA. A total of 100 ng of nucleic acid from each replicate were retrotranscribed (first strand synthesis) and labelled using a Low Input Quick Amp Labeling kit (Agilent) following manufacturer protocols for two-colour, microarray-based, gene expression analysis. Arabidopsis (V4) gene expression microarrays covering 4 × 44 K probes (Agilent microarray design ID 021169, P/N G2519F-021169) were used for subsequent hybridisations in a quadruplicate design in which each exposure condition was labelled in one colour and compared with the unexposed control in the other colour. Hybridised microarrays were scanned on a G2565CA DNA scanner (Agilent). Microarray hybridisation and feature extraction, as well as RNA quality report and labelling, were performed according to Agilent standard procedures and software using the Gene Expression Unit of the Genomics and Proteomics Facility in the UPV/EHU (SGIker platforms; Leioa, Spain). Feature-Extraction-generated files were the input .txt files used to produce normalized signals in GeneSpring GX V12.6 software (GeneLevel_TwoColor_21169_577087196; Agilent).

Normalised microarray data from the 43,663 oligo probes that produced detectable signals were reduced to 26,184 gene entities representing the Gene Level experiment that was studied with GeneSpring GX. Two experimental interpretations were created to analyse data. In the *Treatment* interpretation, 16 conditions were created corresponding to each of the samples exposed to NPs (**AgNP10**, **AgNP20**, **AgNP40**, **AgNP80**, **TiO**_**2**_**NP10**, **TiO**_**2**_**NP 20**, **TiO**_**2**_**NP40**, and **COOH-MWCNT**), bulk materials (**AgNO**_**3**_ and **TiO**_**2**_), biotic (**Pst** or **Abr**) or abiotic (**NaC**, **Drou** and **Wou**) stresses, and ABA treatment (**COOH-MWCNT+**). PCA, GO, and clustering analysis were performed under this interpretation. To identify the set of 351 NP-responsive genes, the eight NP-exposed samples were grouped together into one experimental condition for the second interpretation (*NP addition: Yes* vs. *No*) and analysed with the Statistical Analysis tool (Agilent) using a *t*-test against zero and the Benjamini-Hochberg false discovery rate (FDR) multiple testing correction. The significance threshold for fold-change expression was set to fold change > 2 and FDR < 0.05. For the subsets of 141, 114, 34, 16, and 0 genes responding to **Abr**, **Pst**, **NaC**, **Drou**, and **Wou** stresses, independent *t*-tests were performed for each condition under the *Treatment* interpretation. This produced five independent sets of stress-responsive genes from the total of 26,184 entities; Venn diagrams were then used to obtain the five subsets within the group of 351 NP-responsive genes. GO analysis was performed as implemented in GeneSpring with a cut-off *p*-value < 0.05. Supplemental data were exported from GeneSpring under the corresponding interpretation as .txt files. Box-plots were represented from exported values using Excel (Microsoft, Redmond, WA, USA).

### RT-qPCR

Primers for RT-qPCR (Additional file 7) were validated in a 7900HT Fast Real Time System (Applied Biosystems, Foster City, CA, USA). Total RNA from plants was obtained as in the microarray experiments, and 100 ng were treated with DNAse I (Invitrogen, Carlsbad, CA, USA), retrotranscribed using the qScript cDNA synthesis kit (QUANTA BioSciences, Gaithersburg, MD, USA) and preamplified with QIAGEN® Multiplex PCR Kit (Venlo, Netherlands). Fast Gene Expression Analysis was performed by the SGIker platform using EvaGreen dye (Bio-Rad, Hercules, CA, USA) on a BioMark HD nanofluidic qPCR system (Fluidigm, South San Francisco, CA, USA) following the manufacturer recommendations. Data from quadruplicate runs were analysed with Fluidigm Real-Time PCR Analysis Software V3.1.3 and GenEx V5.4 (MultiD). The average signals of target genes were normalised with respect to the geometric mean from three endogenous control genes (*ACT8*, *TPK2*, and *ADK2*) and relative expression (log scale) for each gene was calculated as the ΔCt between untreated and treated plants. Data presented in figures are average relative expressions and standard errors of the mean (SEM) from three biological replicates.

### *Pst* infection and titering in leaves of NP-exposed plants

Series of eight plants were infected with the *Pst* strain 24 h after exposure to TiO_2_NPs (20 nm size). Infection was performed by inoculating 10^−3^ ODs from a stationary-stage culture or a 10 mM MgSO_4_ mock solution into the major leaf of each plant. Infected plants were collected 2 days later, and the major leaf and its opposite (distal) leaf were cut with a sterile scalpel and introduced into small Petri dishes to quantify the leaf surface under a Zeiss Stemi 2000-C stereo microscope (Jena, Germany). Leaves were homogenized in 400 μL of PBS and 200 μL of *ballotini* (Sigma–Aldrich) with a small pestle, and serial dilutions of the supernatant were plated on selective KB medium (2% protease peptone, 0.15% MgSO_4_-7H_2_O, 0.2% KH_2_PO_4_, 1% glycerol, 1.2% agar, 25 μg/μL Rifampicin). Plates were incubated at 22°C for 24 h, then *Pst* colony-forming units were counted in triplicate plates. A control experiment to determine the effect of NPs on bacterial growth was performed by inoculating bacteria in NP-added or non-added MS medium in the absence of the plant. In this experiment, treatment with 20 nm TiO_2_NPs did not affect bacterial titre.

### Root hair quantification

To quantify the number of root hairs, series of eight plants per treatment were fixed in a H_2_O/ethanol/acetic acid (4:3:1) plus 0.005% Tween 20 solution 4 days after NP treatment. Lateral roots were dissected under a Zeiss stereomicroscope and mounted on microscope slides in a 10% glycerol solution. Slides were observed under the 4× objective of a Nikon Eclipse E 400 microscope (Tokyo, Japan) under transmitted light. Pictures of at least 2 cm of root length and of five apical root segments per plant were taken with a digital AxioCam ERc-5 s (Carl Zeiss, Germany), and the numbers of hairs in the differentiation zones were counted using Zeiss ZEN 2012 software.

### Availability of supporting data

The data sets supporting the results of this article are included within the article and its additional files. Microarray hybridisation data have been submitted to ArrayExpress (EMBL-EBI-European Bioinformatics Institute) (https://www.ebi.ac.uk/arrayexpress/) under the accession number E-MTAB-3331.

## Additional files

Additional file 1:
**Table containing normalized expression values for the 26,184 genes.**
Additional file 2:
**Table listing the 351 differentially-expressed genes in response to NP treatments.**
Additional file 3:
**Table listing the four subsets of stress-responsive genes.**
Additional file 4:
**Table listing the GO terms and enrichment analyses of the 351 NP-responsive genes.**
Additional file 5:
**Figure depicting the distribution of expression ratios for the three branches of genes in Figure** 5 **.**
Additional file 6:
**Figure showing the clustering view of the genes involved in SAR.**
Additional file 7:
**Figure representing the RT-qPCR quantification of selected genes in the stress, NP, and hormone treatments.**
Additional file 8:
**Figure of the distribution of expression ratios within all of the enriched GO-subsets.**
Additional file 9:
**Figure showing the clustering and distribution of expression ratios of root-hair-cell and non-hair-cell genes.**
Additional file 10:
**Figure depicting the clustering expression view of drought-responsive and hypoxia-related genes.**

## Abbreviations

ABA: Abscisic acid
CNTs: Carbon nanotubes
FDR: False discovery rate
GO: Gene ontology
MJ: Methyl jasmonate
MS: Murashige and Skoog medium
MWCNTs: Multi walled carbon nanotubes
SWCNTs: Single walled carbon nanotubes
NPs: Nanoparticles
PCA: Principal Component Analysis
ROS: Reactive oxygen species
SA: Salicylic acid
SAR: Systemic acquired resistance
