# Supplementary figures and images for: Early response to nanoparticles in the Arabidopsis transcriptome compromises plant defence and root-hair development through salicylic acid signalling
Source: BMC Genomics. 2015 Apr 24;16(1):341. doi: 10.1186/s12864-015-1530-4 (PMC4417227; doi:10.1186/s12864-015-1530-4)

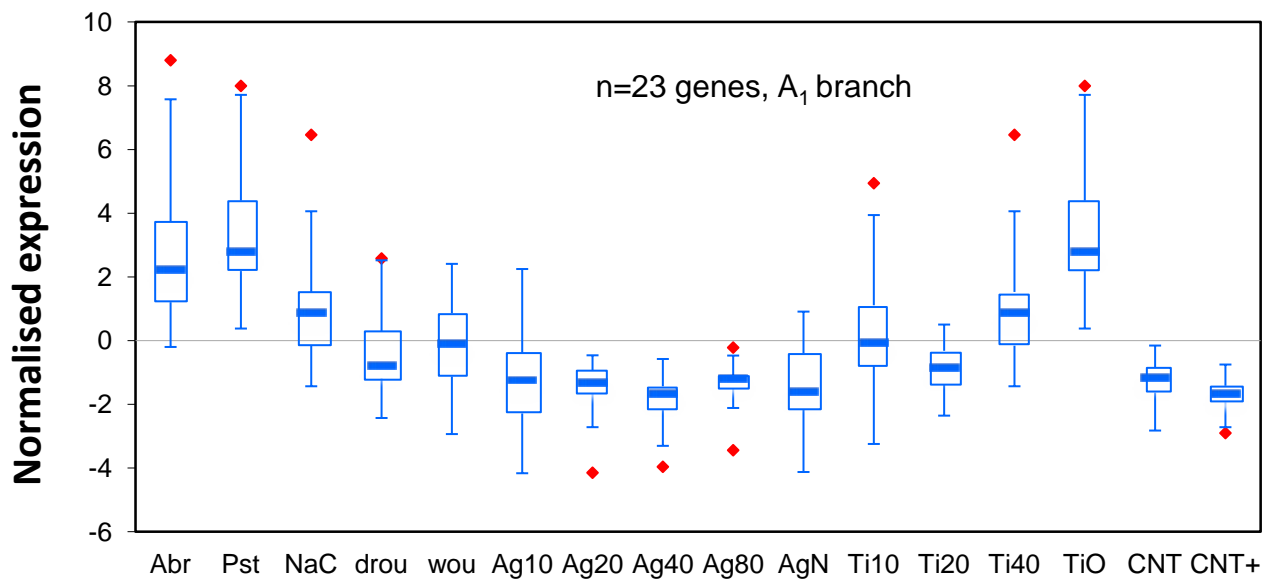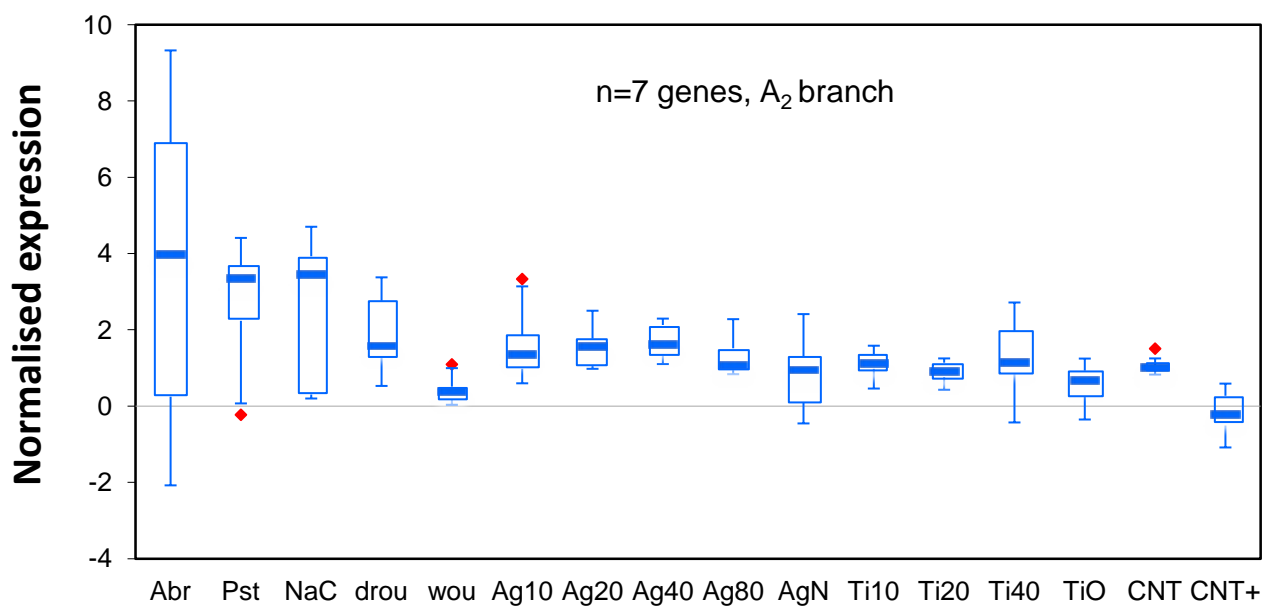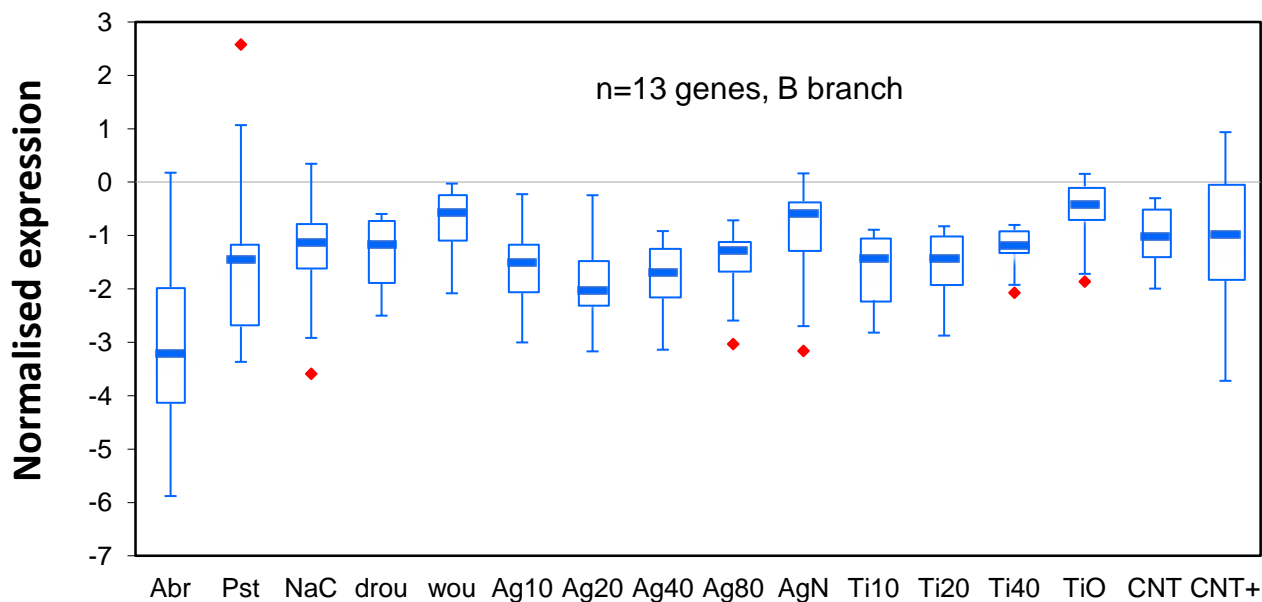

Supplement: Additional file 5: — Figure depicting the distribution of expression ratios for the three branches of genes in Figure 5 . [file 12864_2015_1530_MOESM5_ESM.pdf]

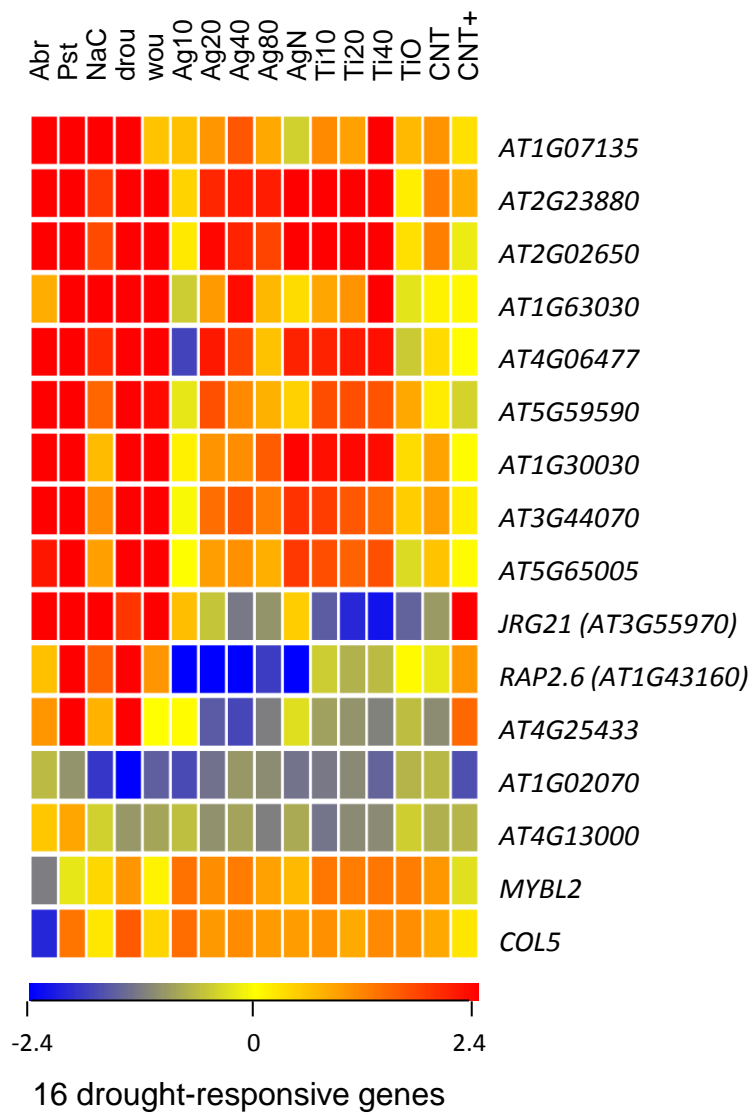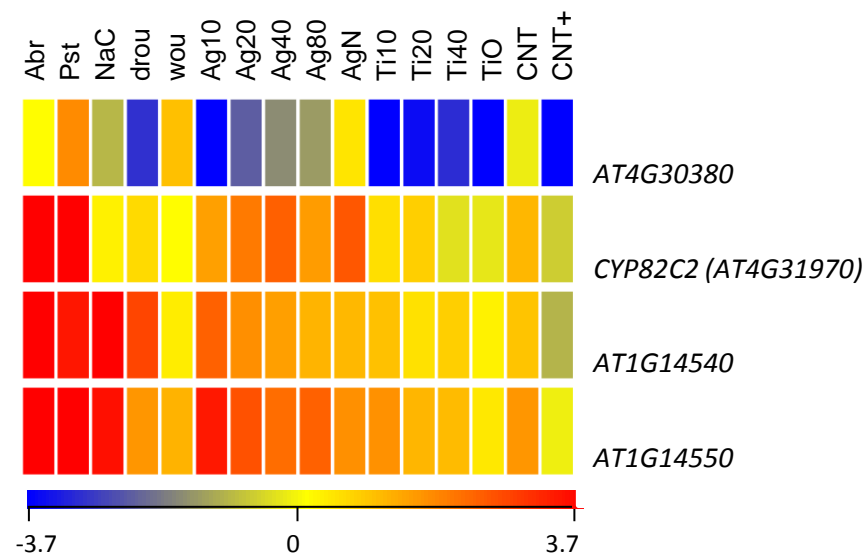

Supplement: Additional file 10: — Figure depicting the clustering expression view of drought-responsive and hypoxia-related genes. [file 12864_2015_1530_MOESM10_ESM.pdf]
