# Supplementary material for: Early response to nanoparticles in the Arabidopsis transcriptome compromises plant defence and root-hair development through salicylic acid signalling
Source: BMC Genomics. 2015 Apr 24;16(1):341. doi: 10.1186/s12864-015-1530-4 (PMC4417227; doi:10.1186/s12864-015-1530-4)
Supplement: Additional file 7: — Figure representing the RT-qPCR quantification of selected genes in the stress, NP, and hormone treatments. [file 12864_2015_1530_MOESM7_ESM.pdf]

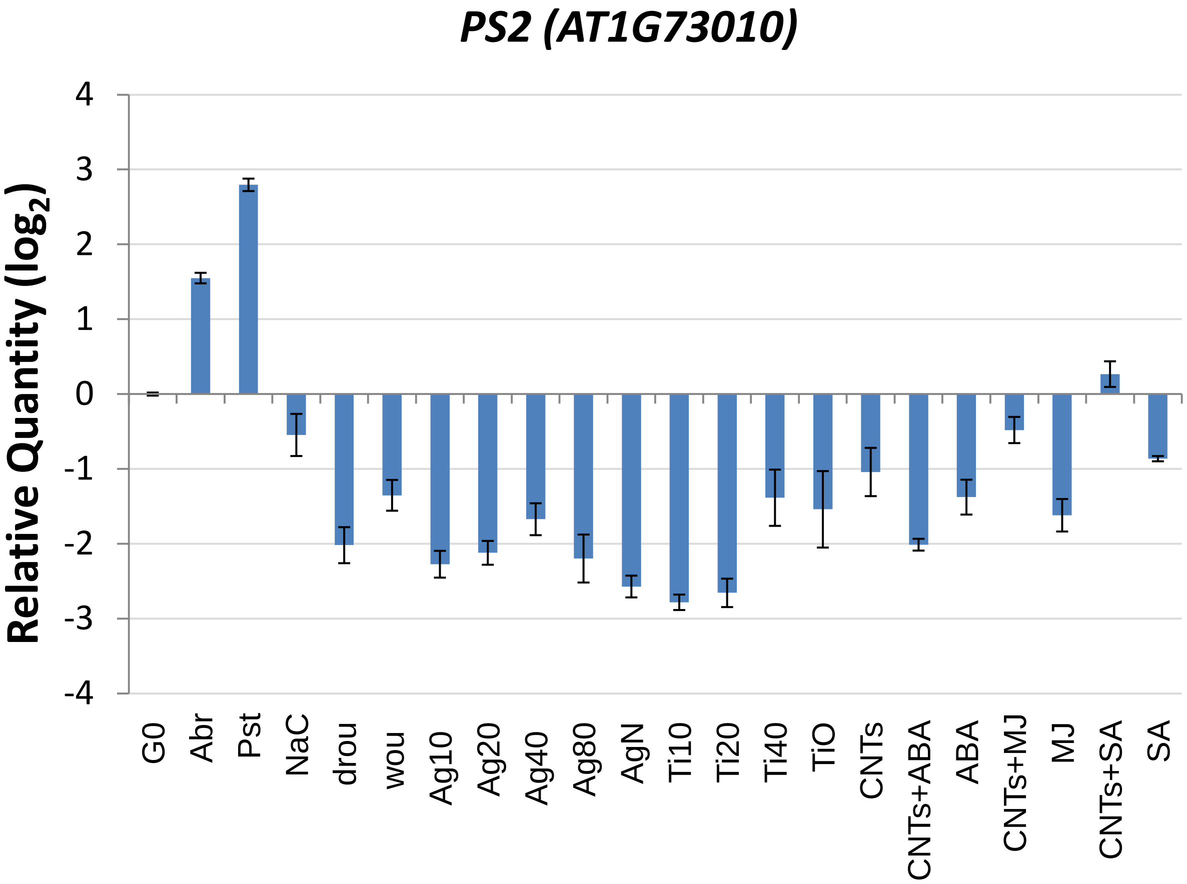

Primers

5’ GCCACGTAAGAATTTCCCTGTA 3´
5’ GCCATCGGTCCATTCTCTAATC 3’

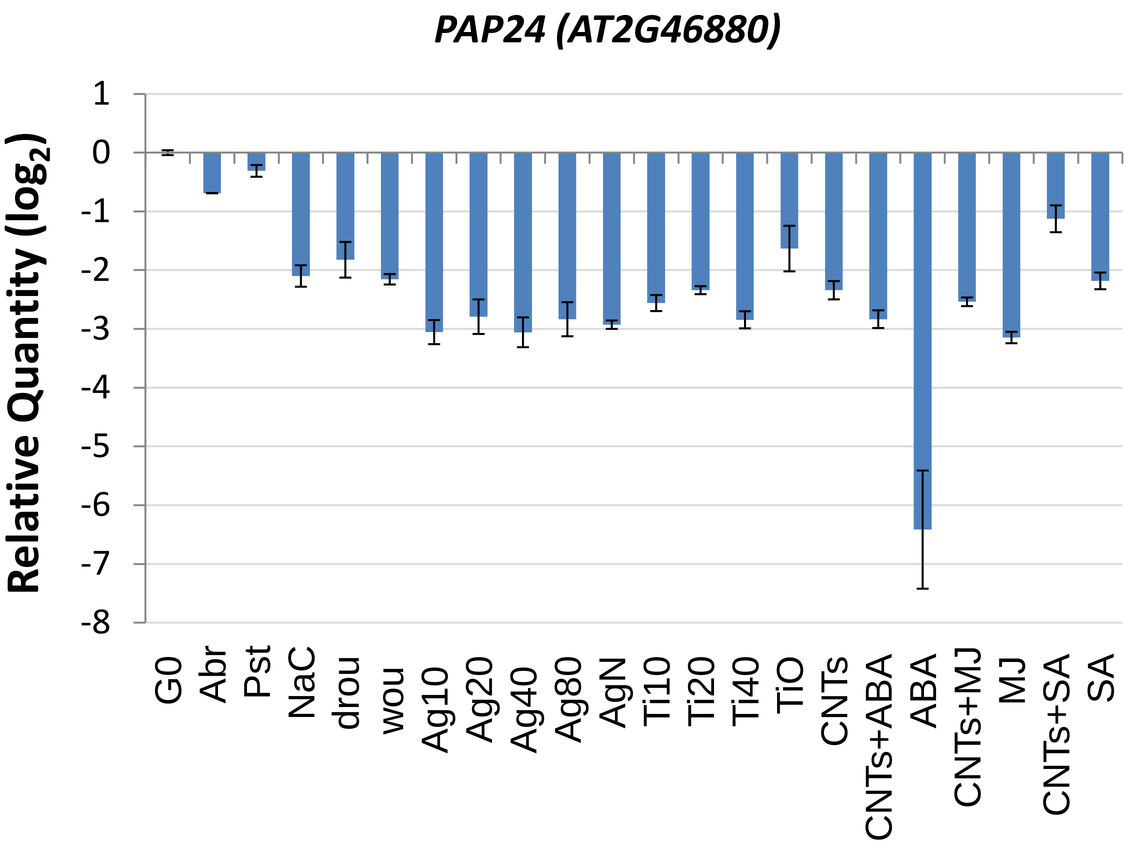

Primers

5’ CACTTGTCTCCCTTTAGCTCAC 3´

5’ CAGTGCTGCTCTTCTTGTACTC 3’

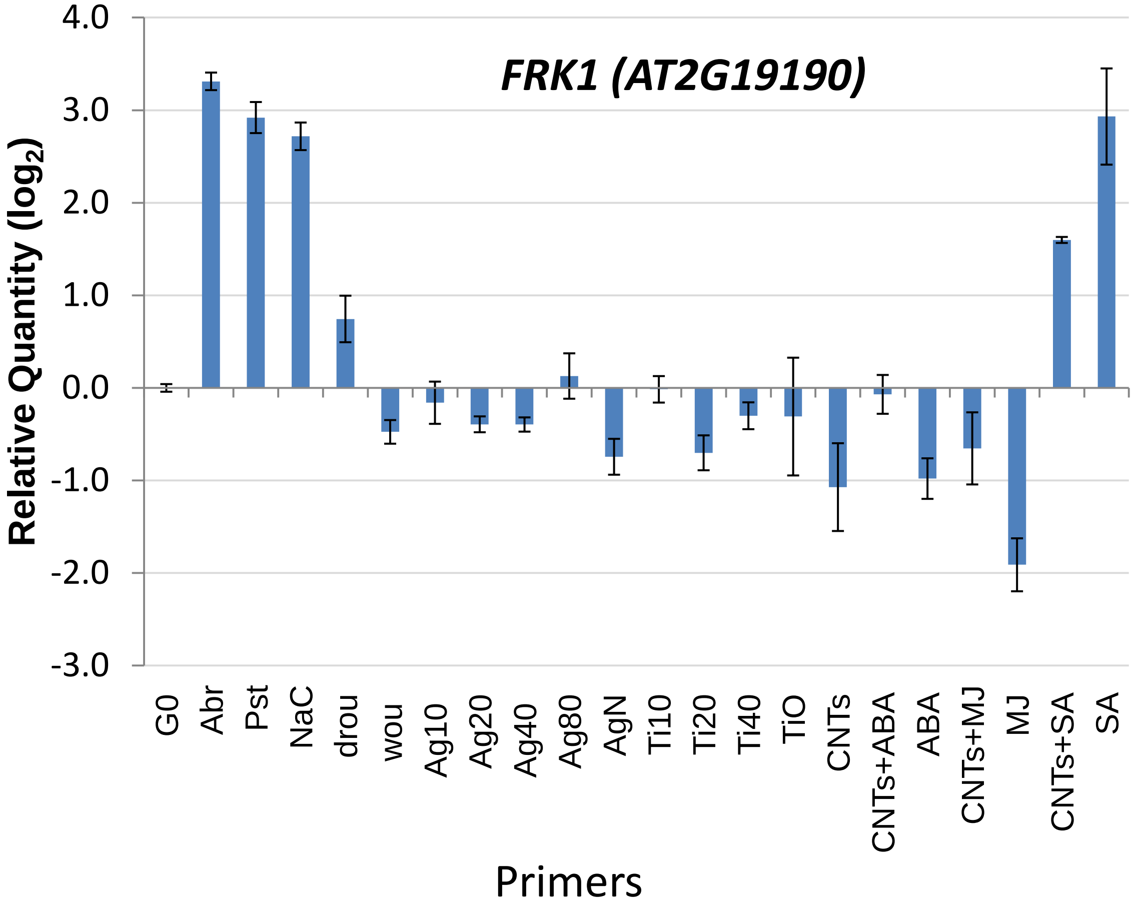

5’ TTACAGGCCAACCTGCTATTG 3´

5’ GCTGATCCACGATTCCTCTAATG 3’

MYBL2 (AT1G71030)

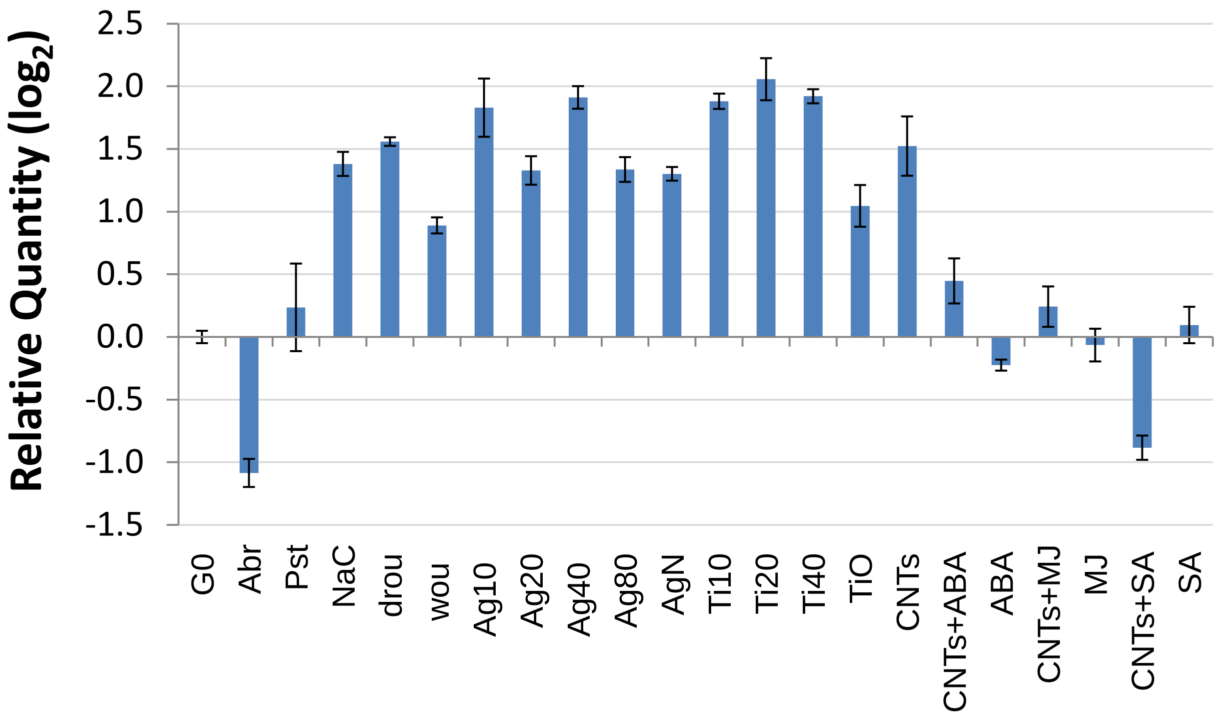

Primers

5’ GACGAACCGACAACGAAGTTAG 3´
5’ GTGATGGTGGAGACGATGATTG 3’

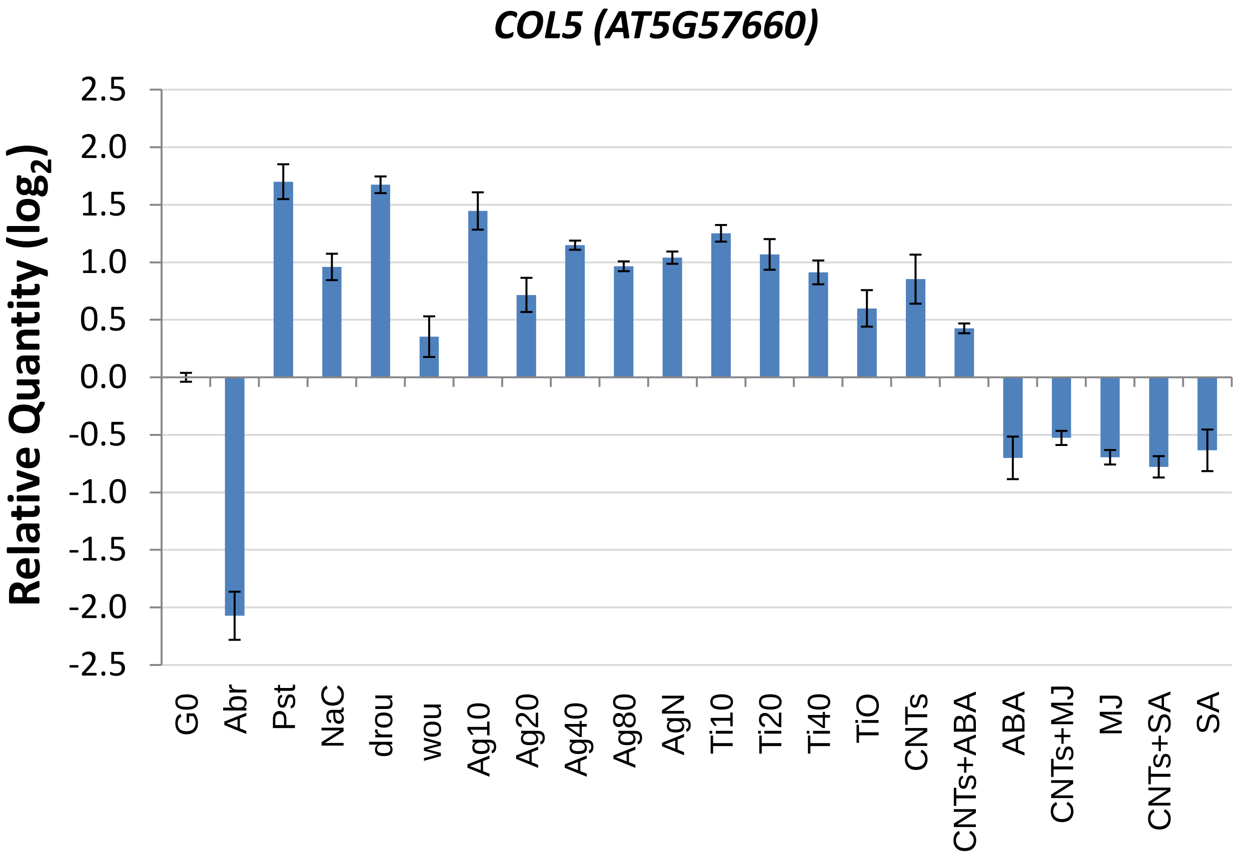

Primers

5’ CAGAGCCTCTCCCGTTAACTA 3´
5’ CTGTGGCTGACTGATTGAGAAG 3’
