# Supplementary material for: Early response to nanoparticles in the Arabidopsis transcriptome compromises plant defence and root-hair development through salicylic acid signalling
Source: BMC Genomics. 2015 Apr 24;16(1):341. doi: 10.1186/s12864-015-1530-4 (PMC4417227; doi:10.1186/s12864-015-1530-4)
Supplement: Additional file 8: — Figure of the distribution of expression ratios within all of the enriched GO-subsets. [file 12864_2015_1530_MOESM8_ESM.pdf]

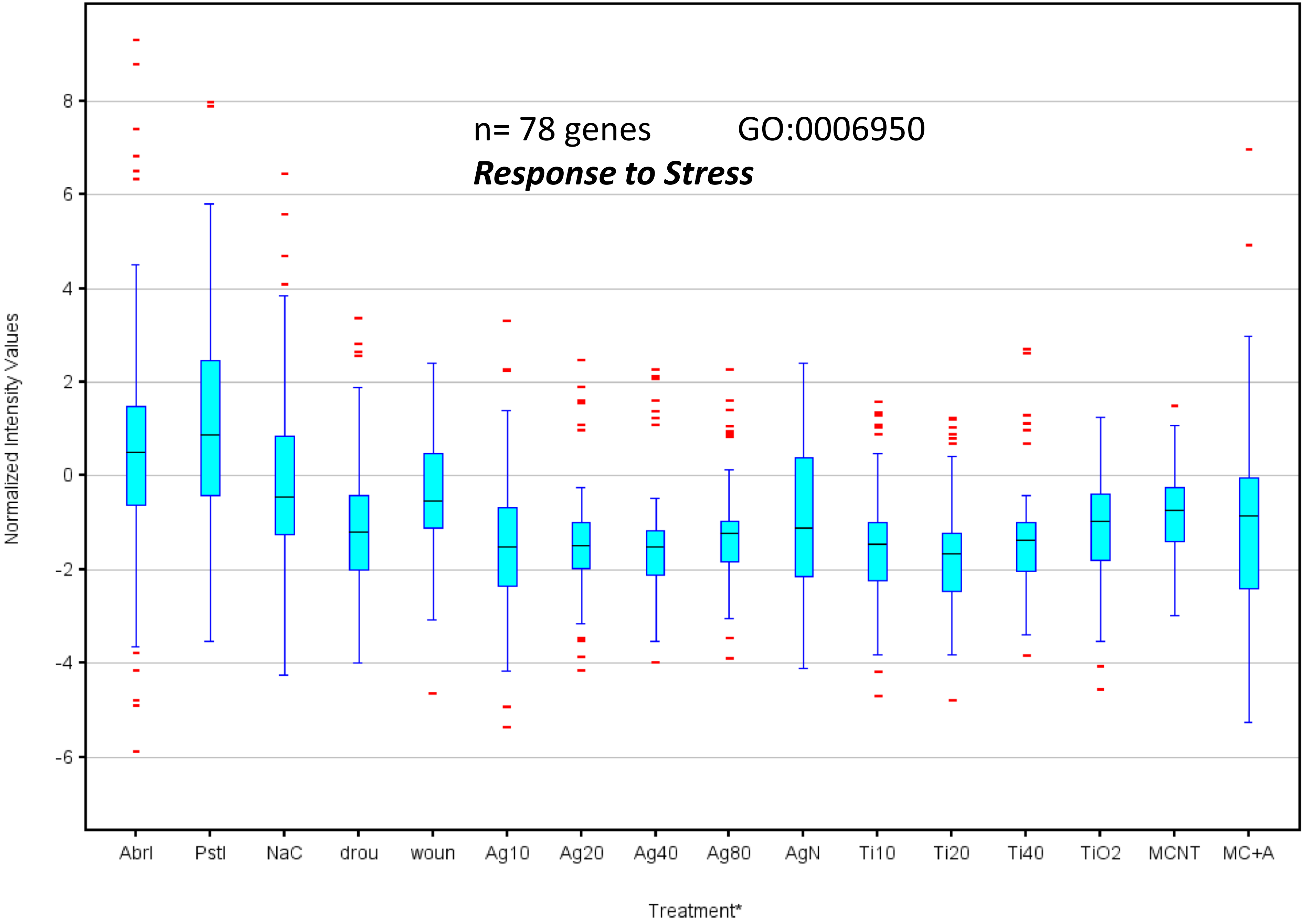

n= 78 genes GO:0006950
*Response to Stress*
Normalized Intensity Values
8
6
4
2
0
-2
-4
-6
Abrl
Pstl
NaC
drou
woun
Ag10
Ag20
Ag40
Ag80
AgN
Ti10
Ti20
Ti40
TiO2
MCNT
MC+A
Treatment*

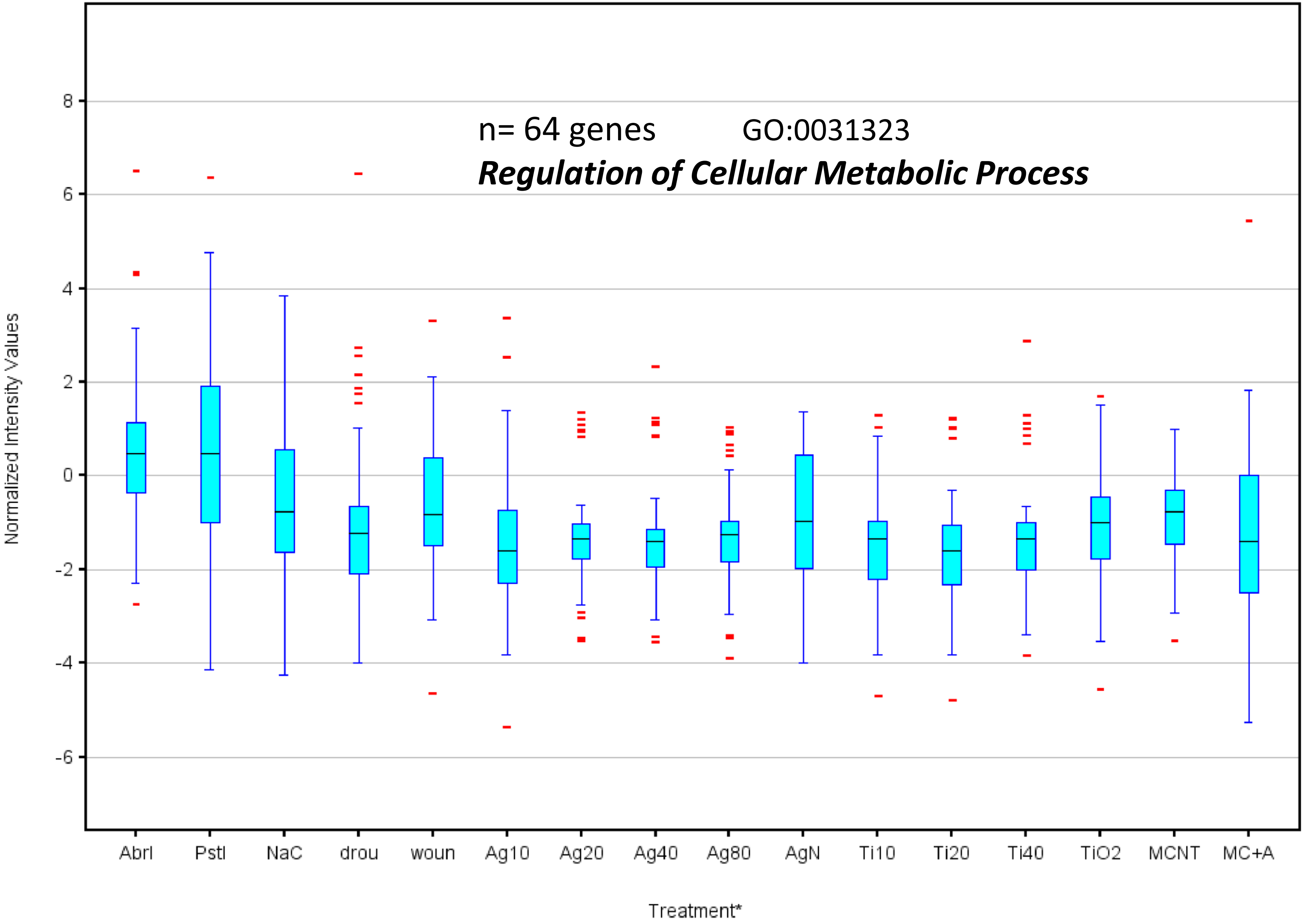

n= 64 genes GO:0031323
Regulation of Cellular Metabolic Process
Normalized Intensity Values
8
6
4
2
0
-2
-4
-6
Abrl
Pstl
NaC
drou
woun
Ag10
Ag20
Ag40
Ag80
AgN
Ti10
Ti20
Ti40
TiO2
MCNT
MC+A
Treatment*

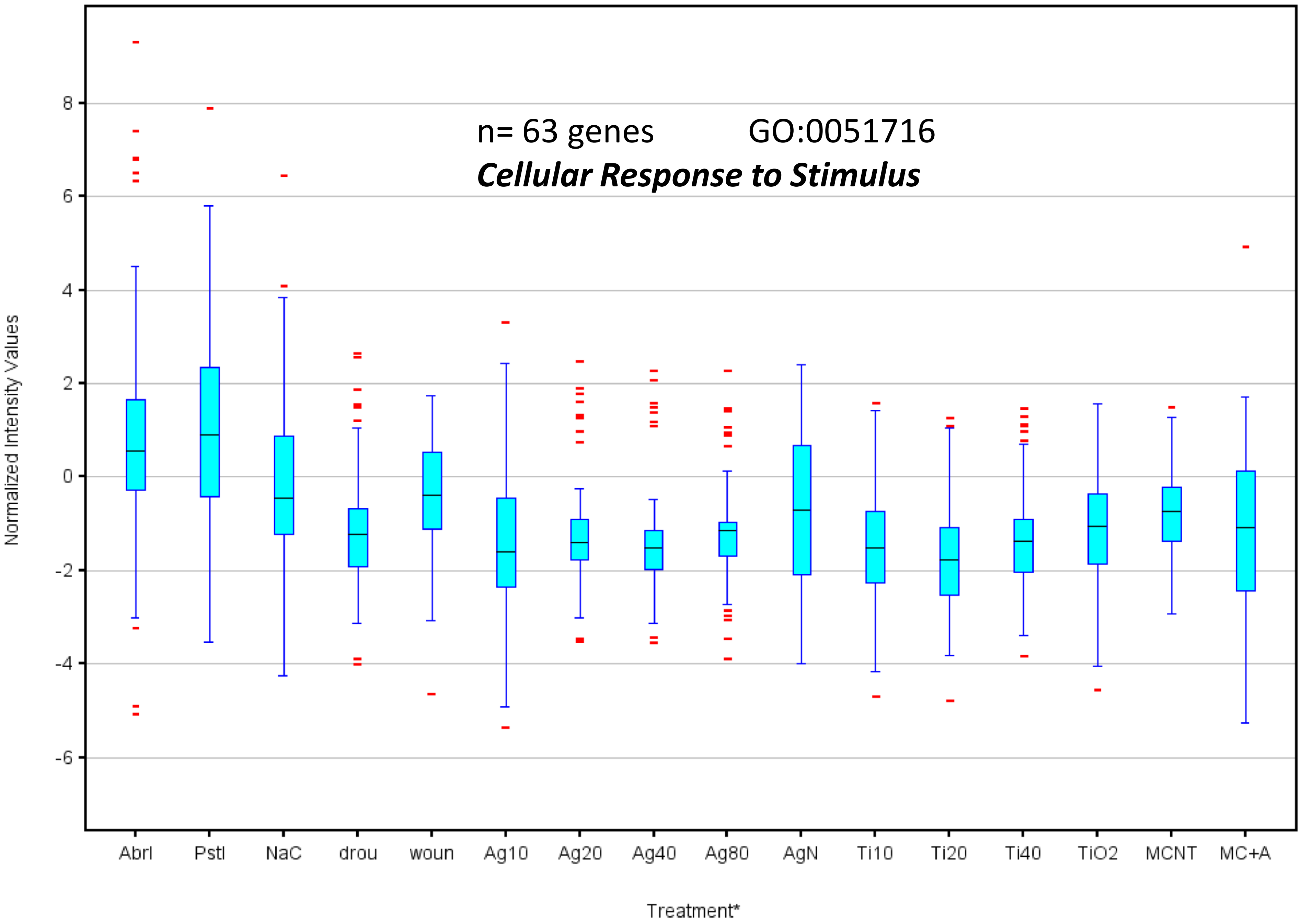
n= 63 genes
GO:0051716
Cellular Response to Stimulus
Normalized Intensity Values
8
6
4
2
0
-2
-4
-6
Abrl
Pstl
NaC
drou
woun
Ag10
Ag20
Ag40
Ag80
AgN
Ti10
Ti20
Ti40
TiO2
MCNT
MC+A
Treatment*

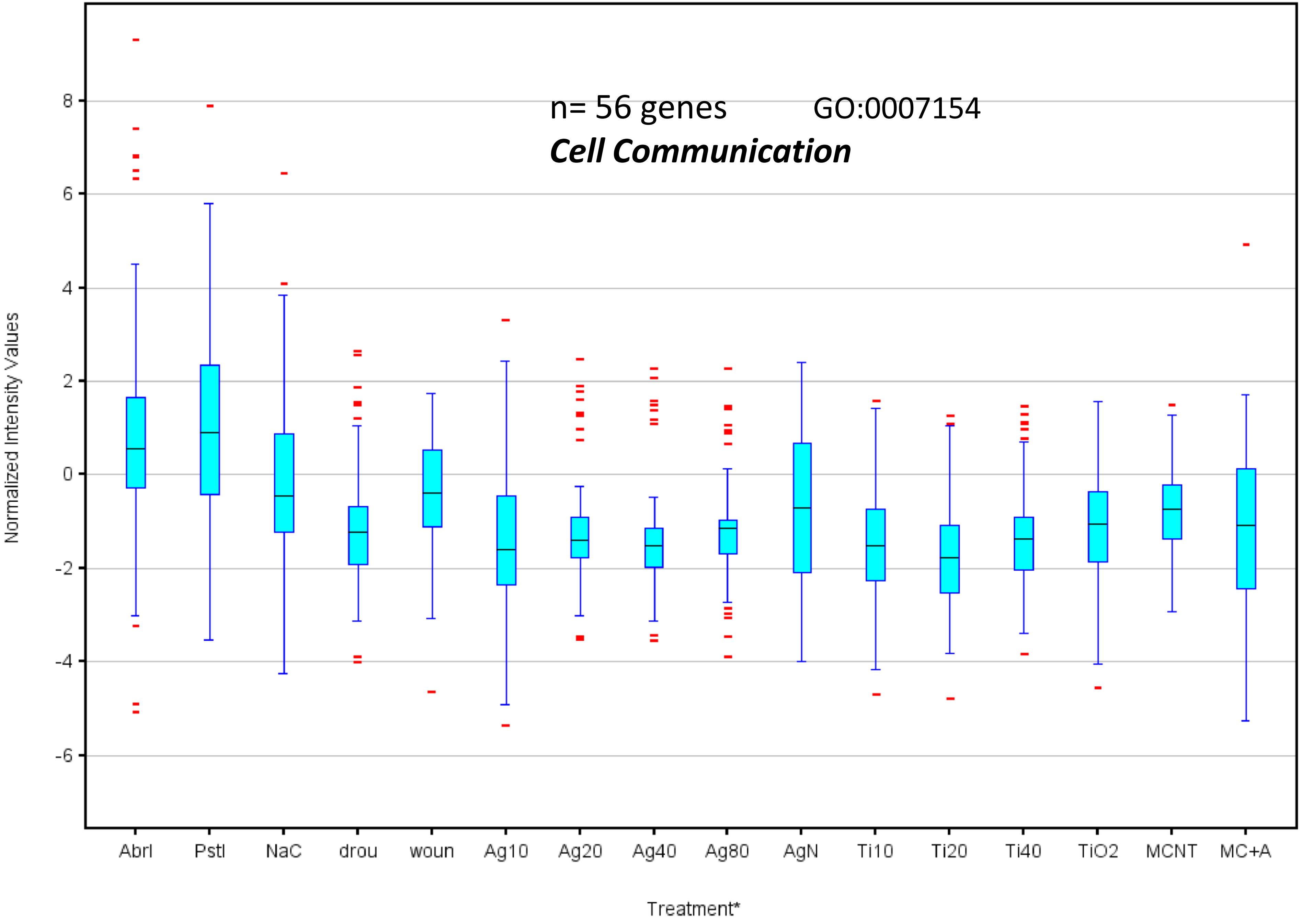

n= 56 genes GO:0007154
*Cell Communication*
Normalized Intensity Values
8
6
4
2
0
-2
-4
-6
Abrl
Pstl
NaC
drou
woun
Ag10
Ag20
Ag40
Ag80
AgN
Ti10
Ti20
Ti40
TiO2
MCNT
MC+A
Treatment*

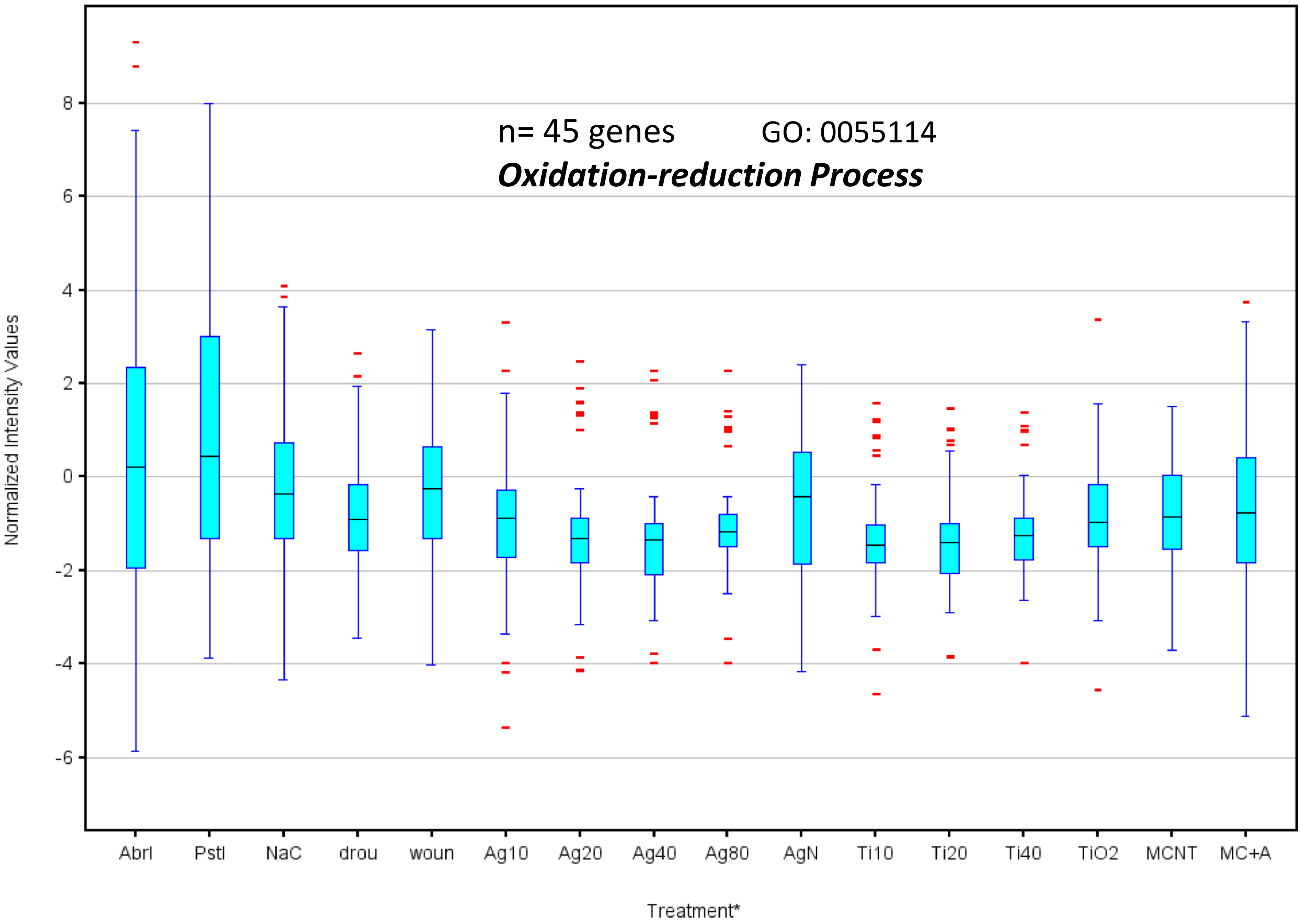
n= 45 genes
GO: 0055114
Oxidation-reduction Process
Normalized Intensity Values
8
6
4
2
0
-2
-4
-6
Abrl
Pstl
NaC
drou
woun
Ag10
Ag20
Ag40
Ag80
AgN
Ti10
Ti20
Ti40
TiO2
MCNT
MC+A
Treatment*

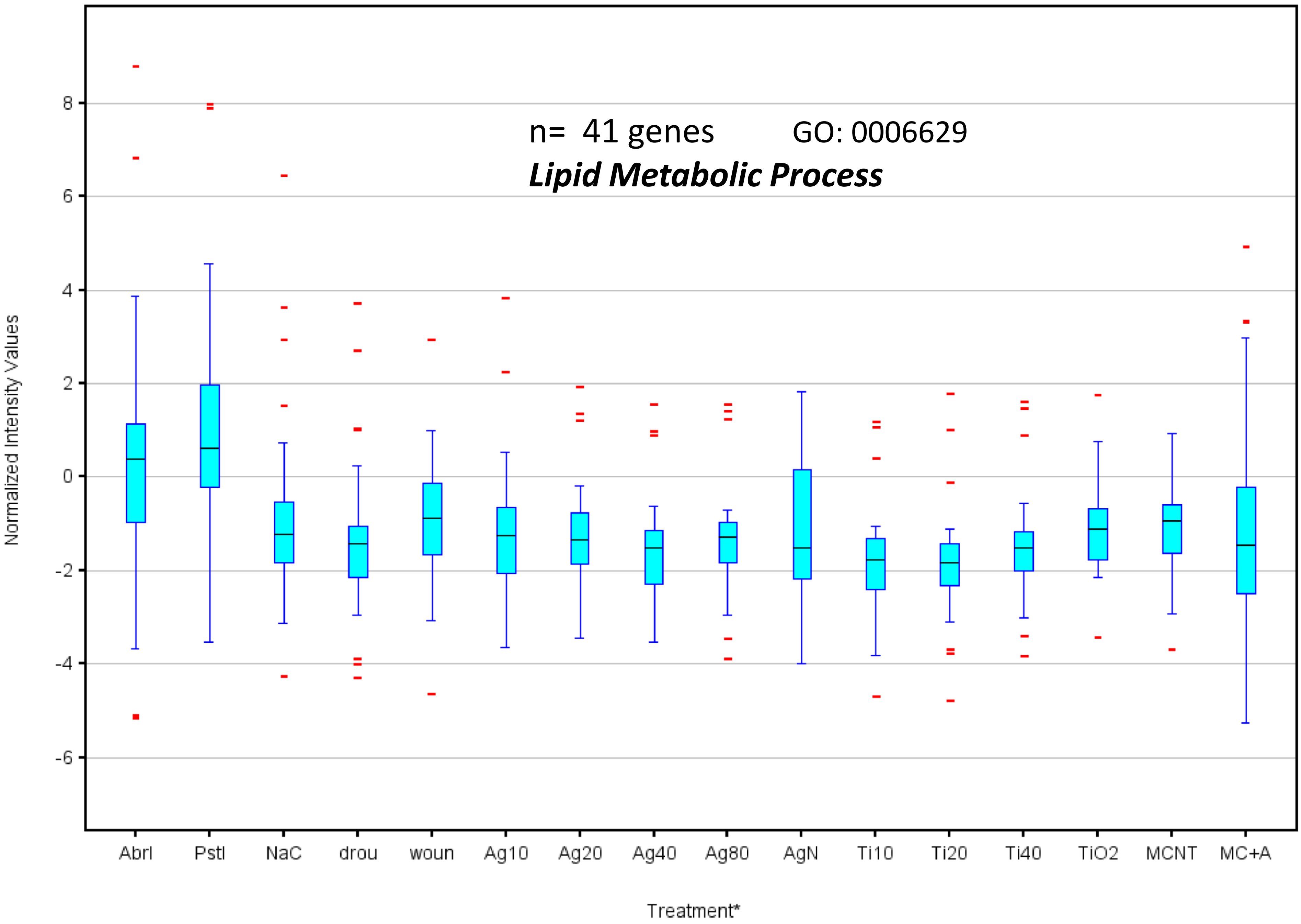

n= 41 genes
GO: 0006629
Lipid Metabolic Process
Normalized Intensity Values
8
6
4
2
0
-2
-4
-6
Abrl
Pstl
NaC
drou
woun
Ag10
Ag20
Ag40
Ag80
AgN
Ti10
Ti20
Ti40
TiO2
MCNT
MC+A
Treatment*

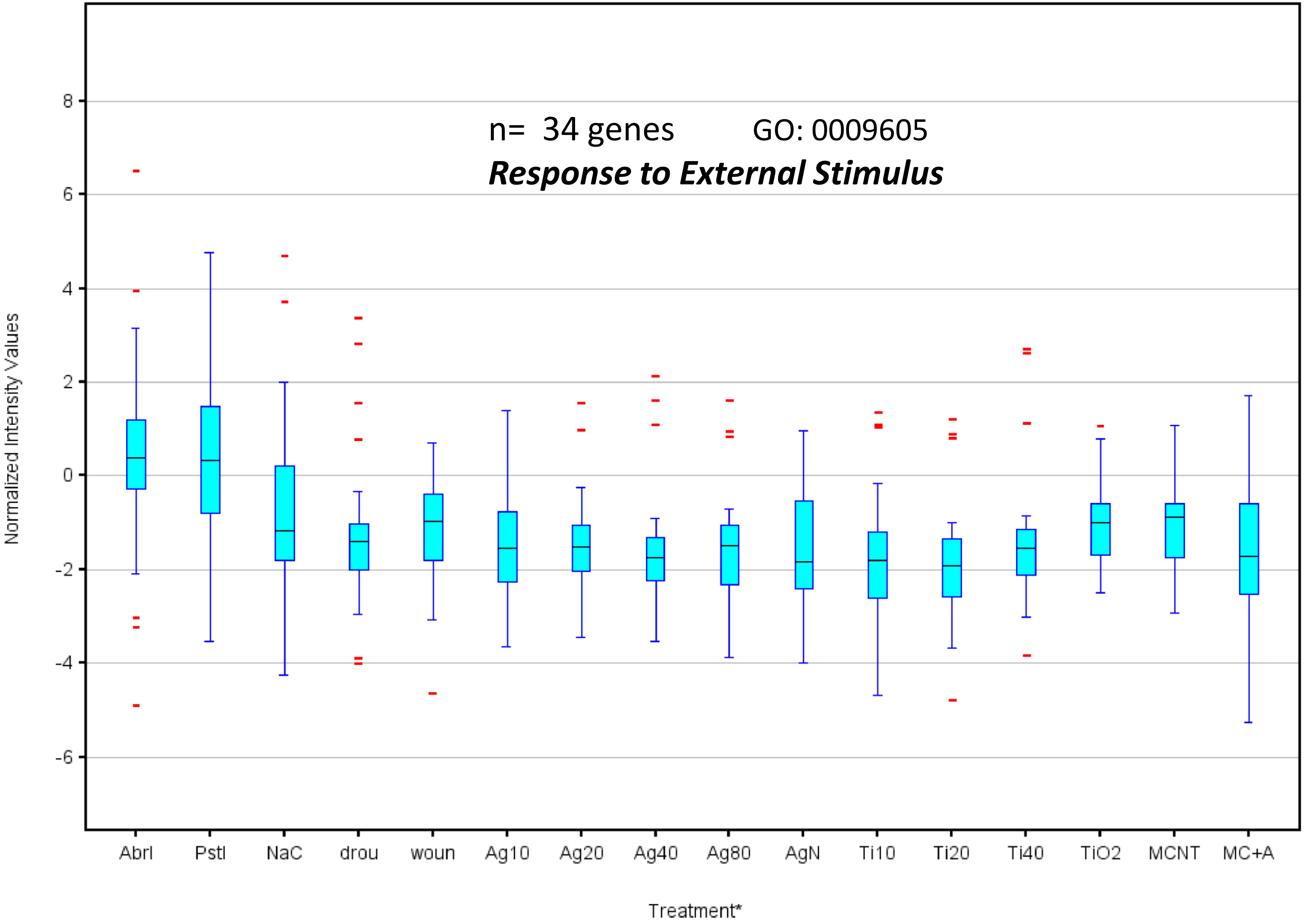

n= 34 genes
GO: 0009605
Response to External Stimulus
Normalized Intensity Values
8
6
4
2
0
-2
-4
-6
Abrl
Pstl
NaC
drou
woun
Ag10
Ag20
Ag40
Ag80
AgN
Ti10
Ti20
Ti40
TiO2
MCNT
MC+A
Treatment*

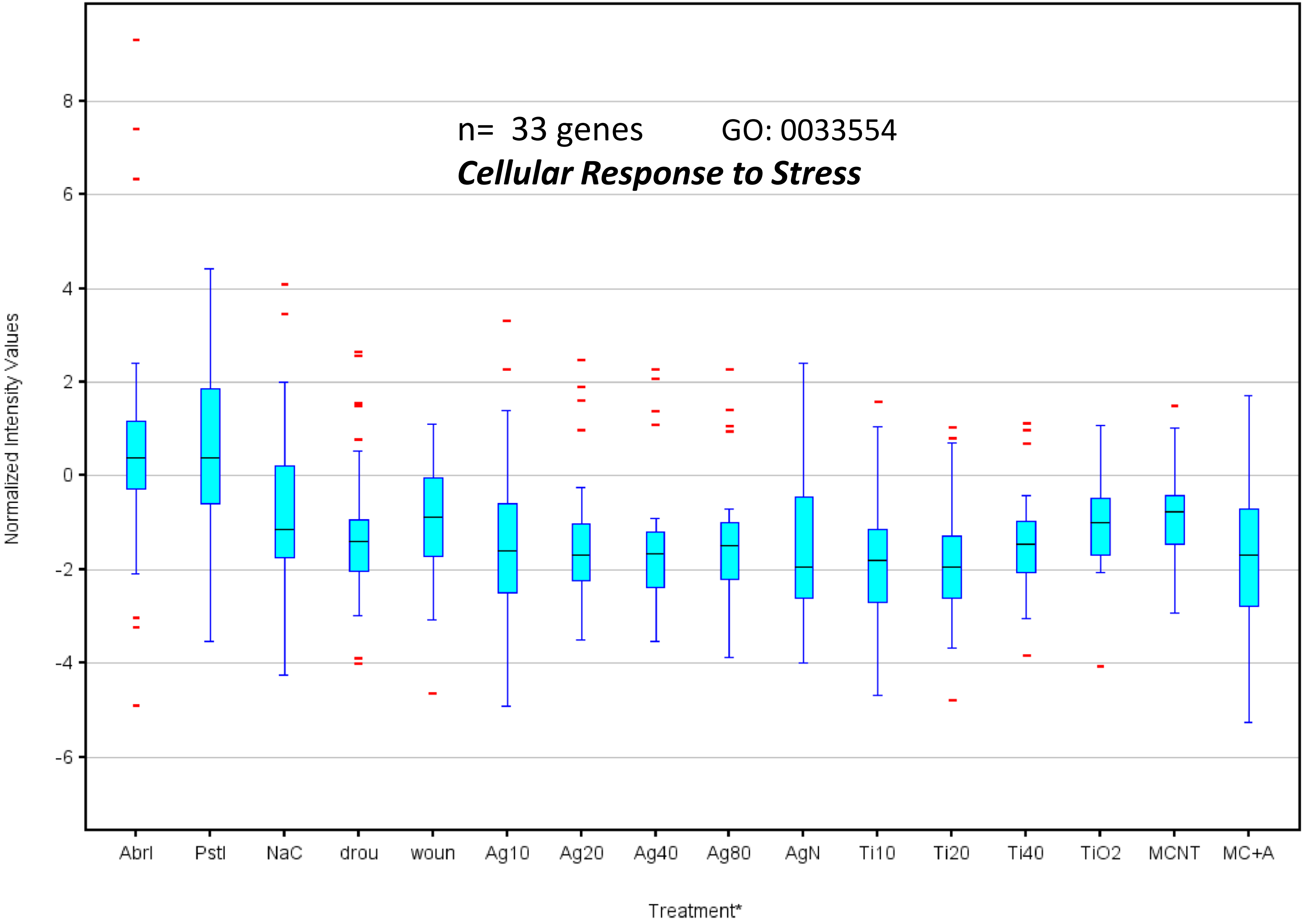

n= 33 genes GO: 0033554
Cellular Response to Stress
Normalized Intensity Values
8
6
4
2
0
-2
-4
-6
Abrl
PstI
NaC
drou
woun
Ag10
Ag20
Ag40
Ag80
AgN
Ti10
Ti20
Ti40
TiO2
MCNT
MC+A
Treatment*

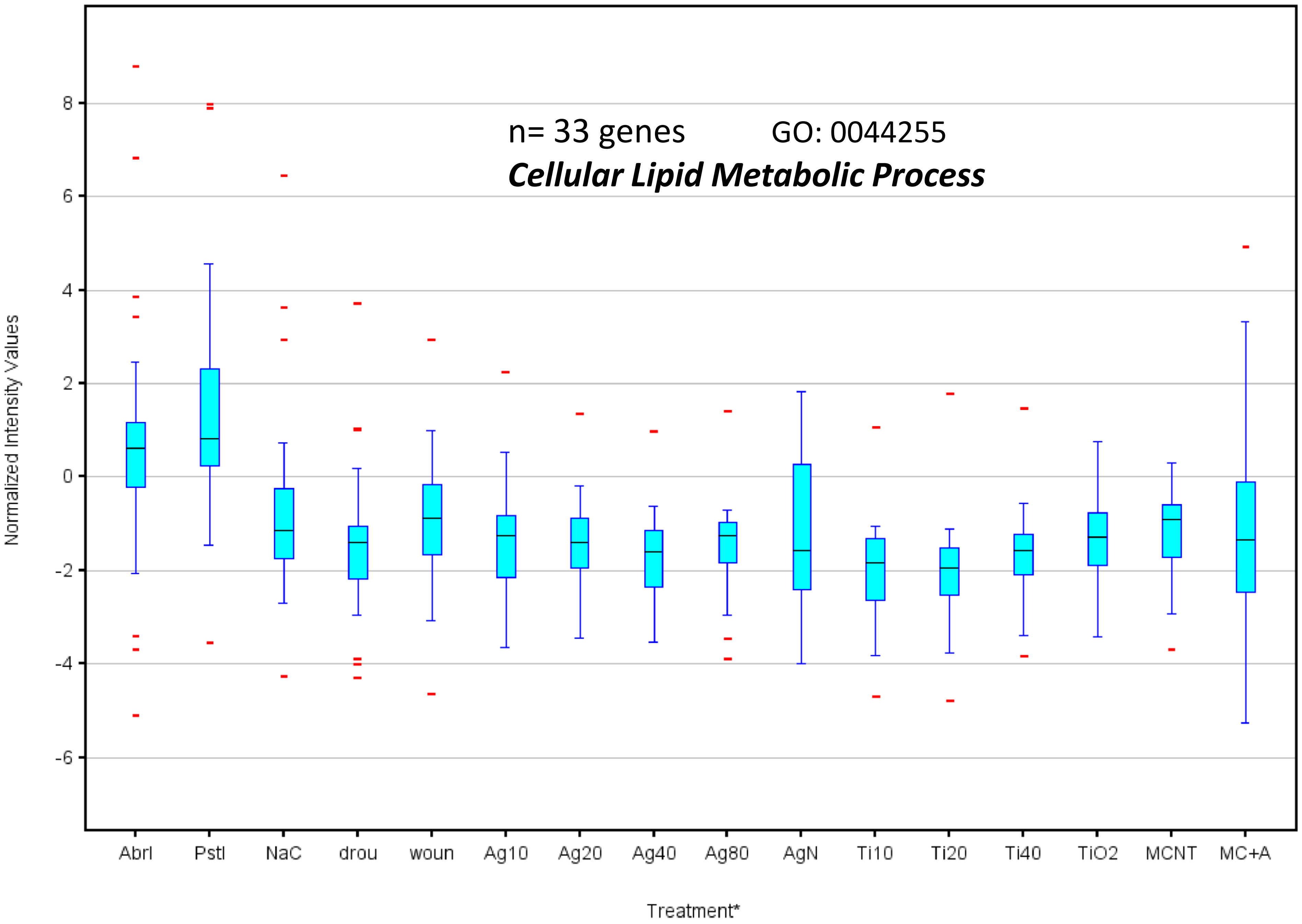

n= 33 genes GO: 0044255
Cellular Lipid Metabolic Process
Normalized Intensity Values
8
6
4
2
0
-2
-4
-6
Abrl
Pstl
NaC
drou
woun
Ag10
Ag20
Ag40
Ag80
AgN
Ti10
Ti20
Ti40
TiO2
MCNT
MC+A
Treatment*

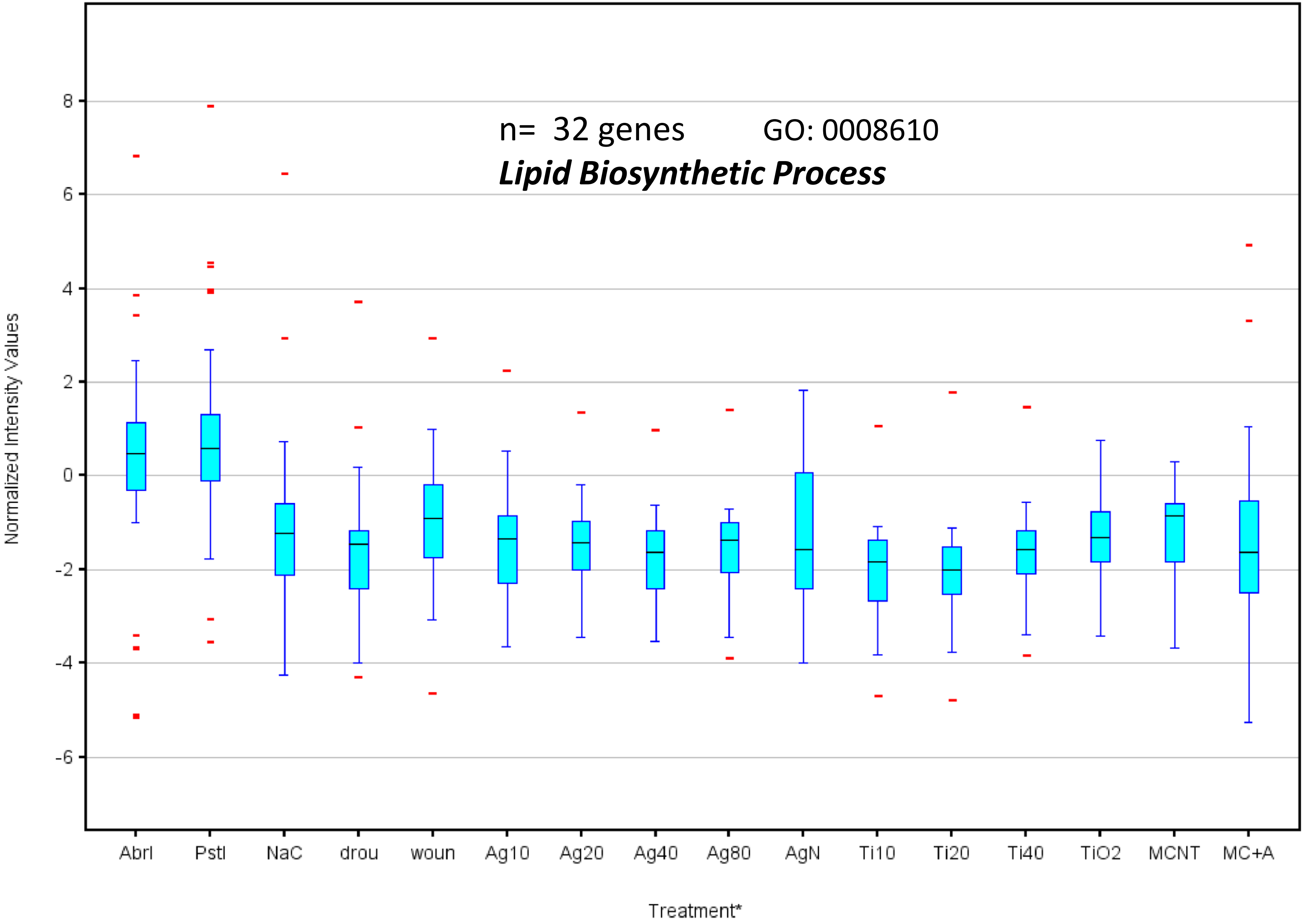

n= 32 genes GO: 0008610
Lipid Biosynthetic Process
Normalized Intensity Values
8
6
4
2
0
-2
-4
-6
Abrl
Pstl
NaC
drou
woun
Ag10
Ag20
Ag40
Ag80
AgN
Ti10
Ti20
Ti40
TiO2
MCNT
MC+A
Treatment*

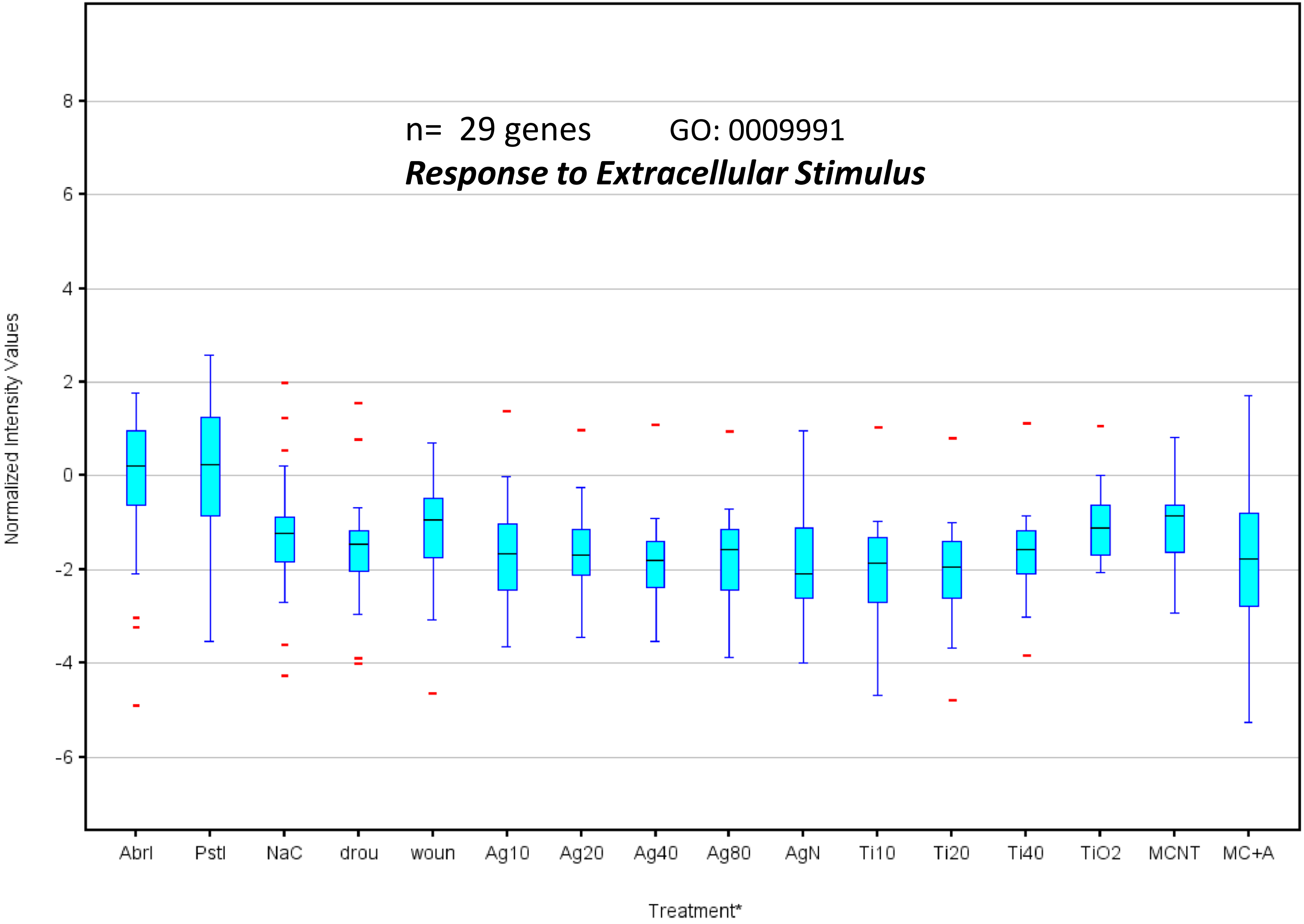
n= 29 genes
GO: 0009991
Response to Extracellular Stimulus
Normalized Intensity Values
8
6
4
2
0
-2
-4
-6
Abrl
Pstl
NaC
drou
woun
Ag10
Ag20
Ag40
Ag80
AgN
Ti10
Ti20
Ti40
TiO2
MCNT
MC+A
Treatment*

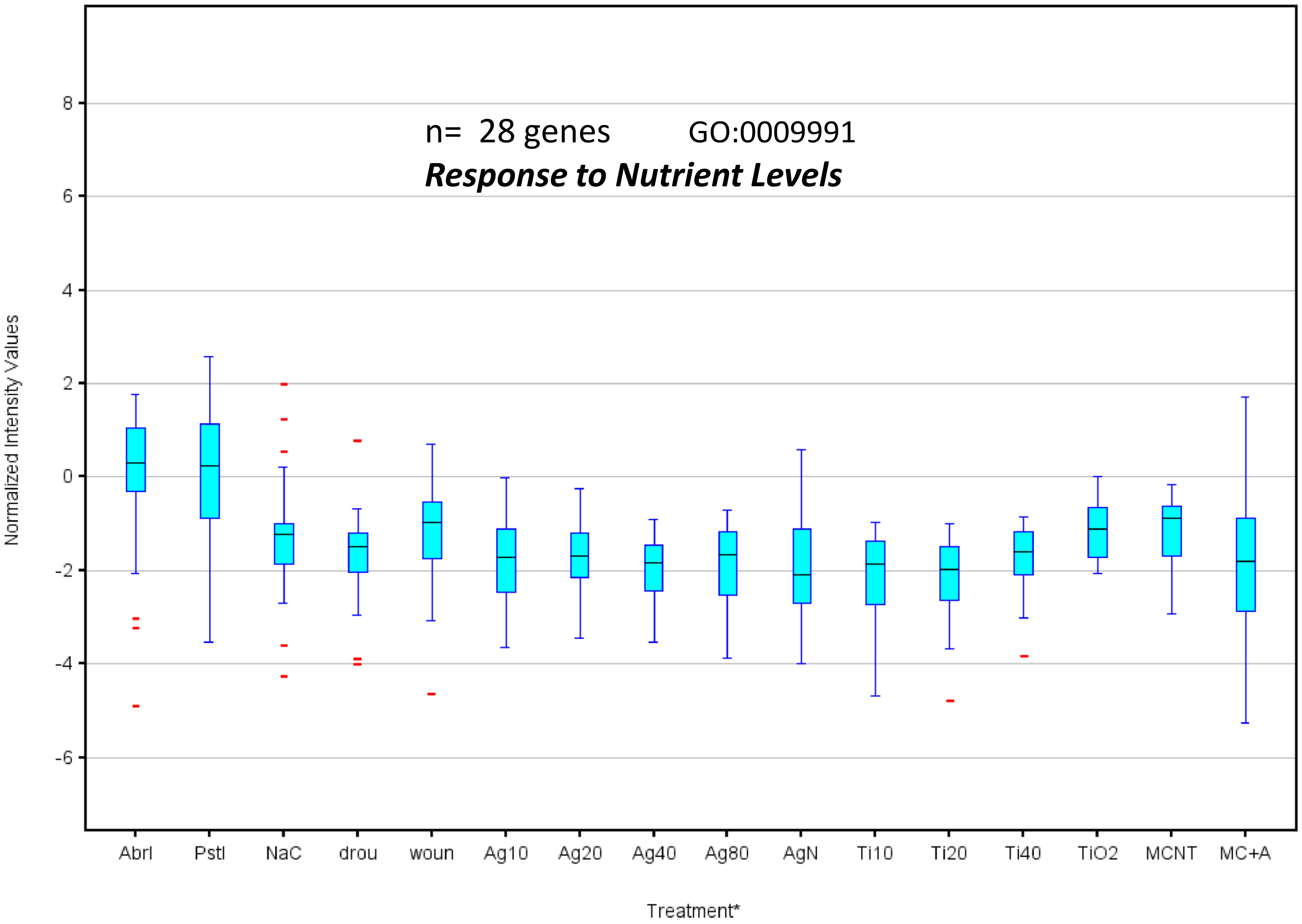

n= 28 genes
GO:0009991
Response to Nutrient Levels
Normalized Intensity Values
8
6
4
2
0
-2
-4
-6
Abrl
Pstl
NaC
drou
woun
Ag10
Ag20
Ag40
Ag80
AgN
Ti10
Ti20
Ti40
TiO2
MCNT
MC+A
Treatment*

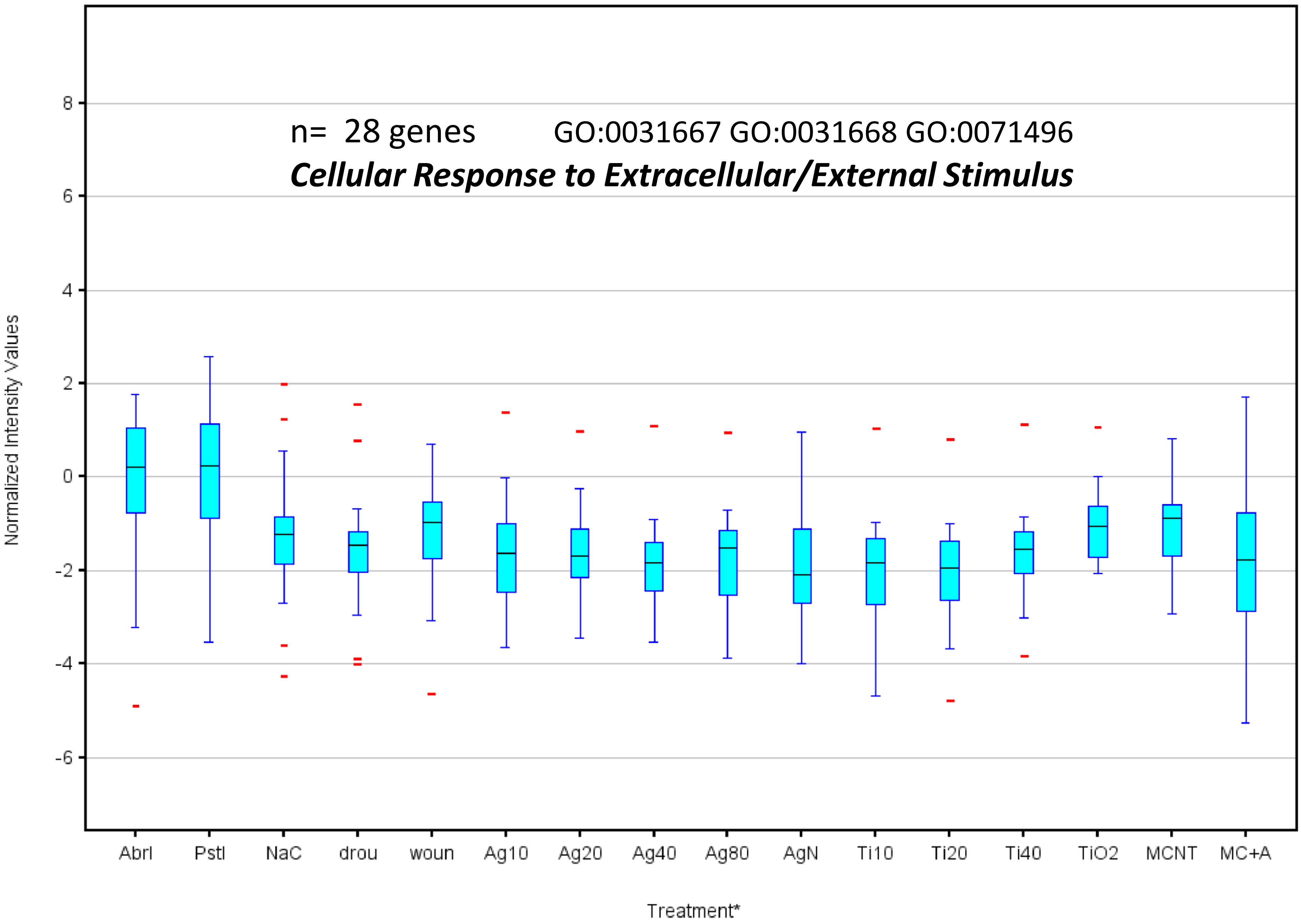
n= 28 genes GO:0031667 GO:0031668 GO:0071496
Cellular Response to Extracellular/External Stimulus
Normalized Intensity Values
8
6
4
2
0
-2
-4
-6
Abrl
Pstl
NaC
drou
woun
Ag10
Ag20
Ag40
Ag80
AgN
Ti10
Ti20
Ti40
TiO2
MCNT
MC+A
Treatment*

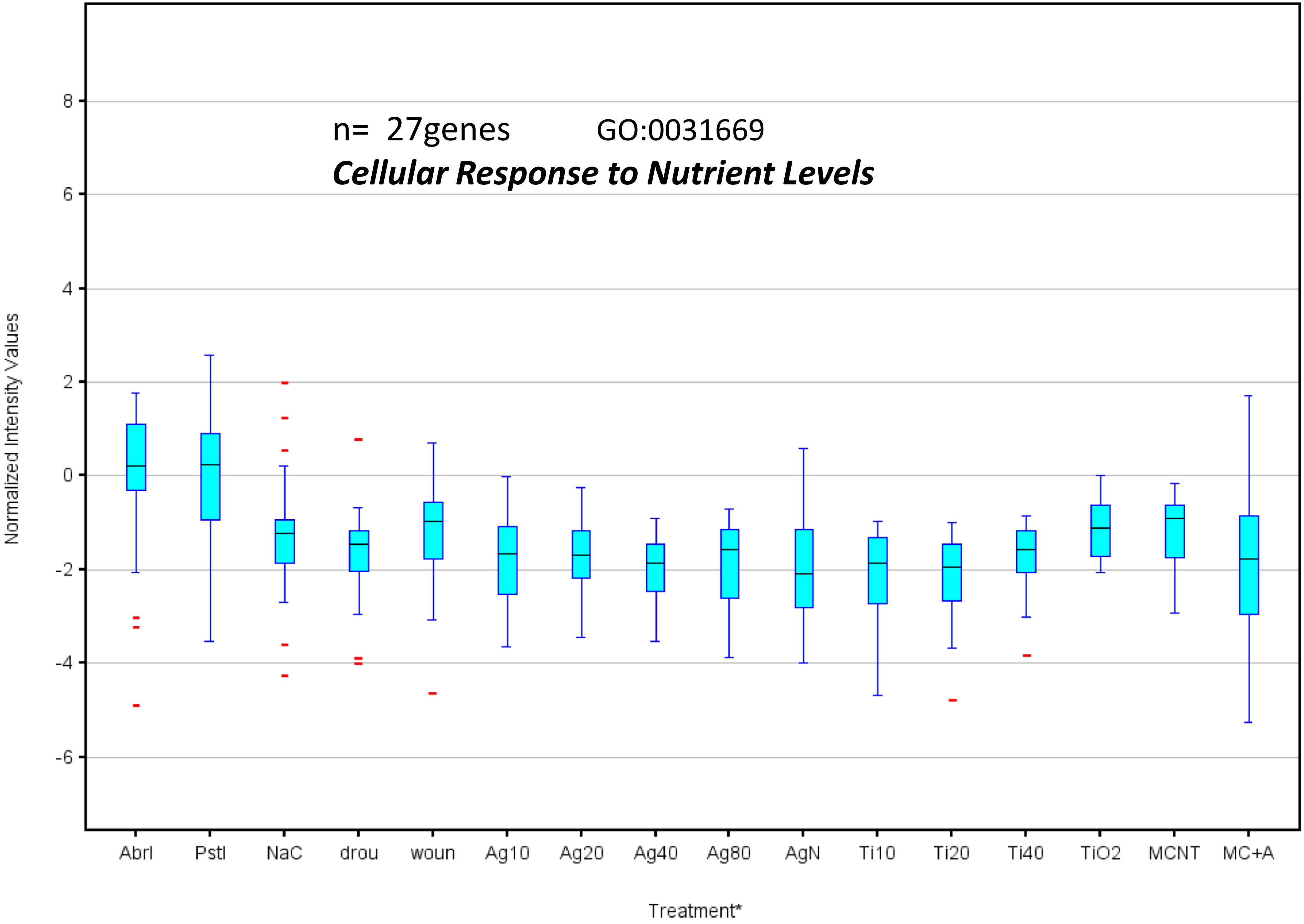

n= 27genes GO:0031669
Cellular Response to Nutrient Levels
Normalized Intensity Values
8
6
4
2
0
-2
-4
-6
Abrl
Pstl
NaC
drou
woun
Ag10
Ag20
Ag40
Ag80
AgN
Ti10
Ti20
Ti40
TiO2
MCNT
MC+A
Treatment*

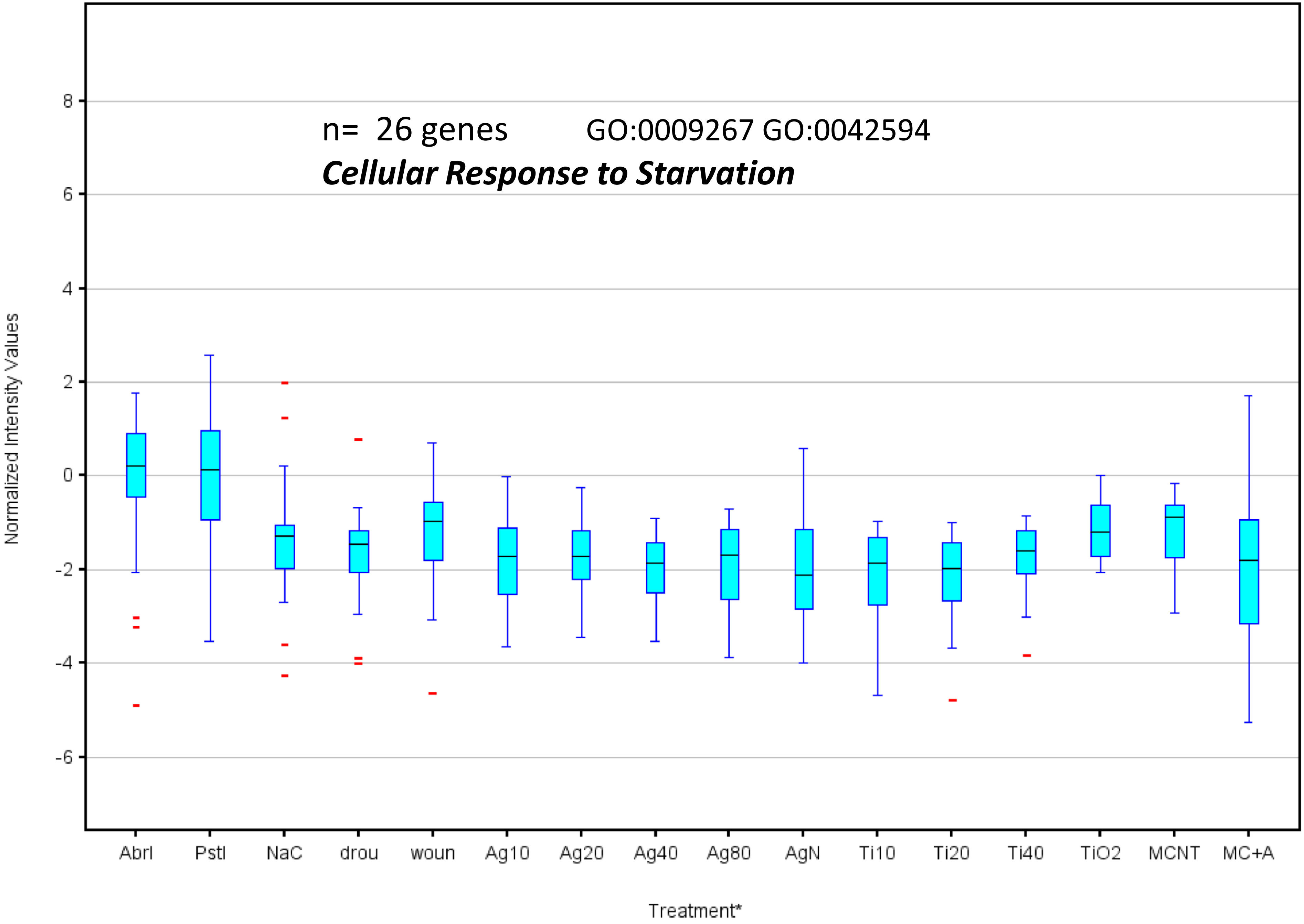
n= 26 genes GO:0009267 GO:0042594
Cellular Response to Starvation
Normalized Intensity Values
8
6
4
2
0
-2
-4
-6
Abrl
Pstl
NaC
drou
woun
Ag10
Ag20
Ag40
Ag80
AgN
Ti10
Ti20
Ti40
TiO2
MCNT
MC+A
Treatment*

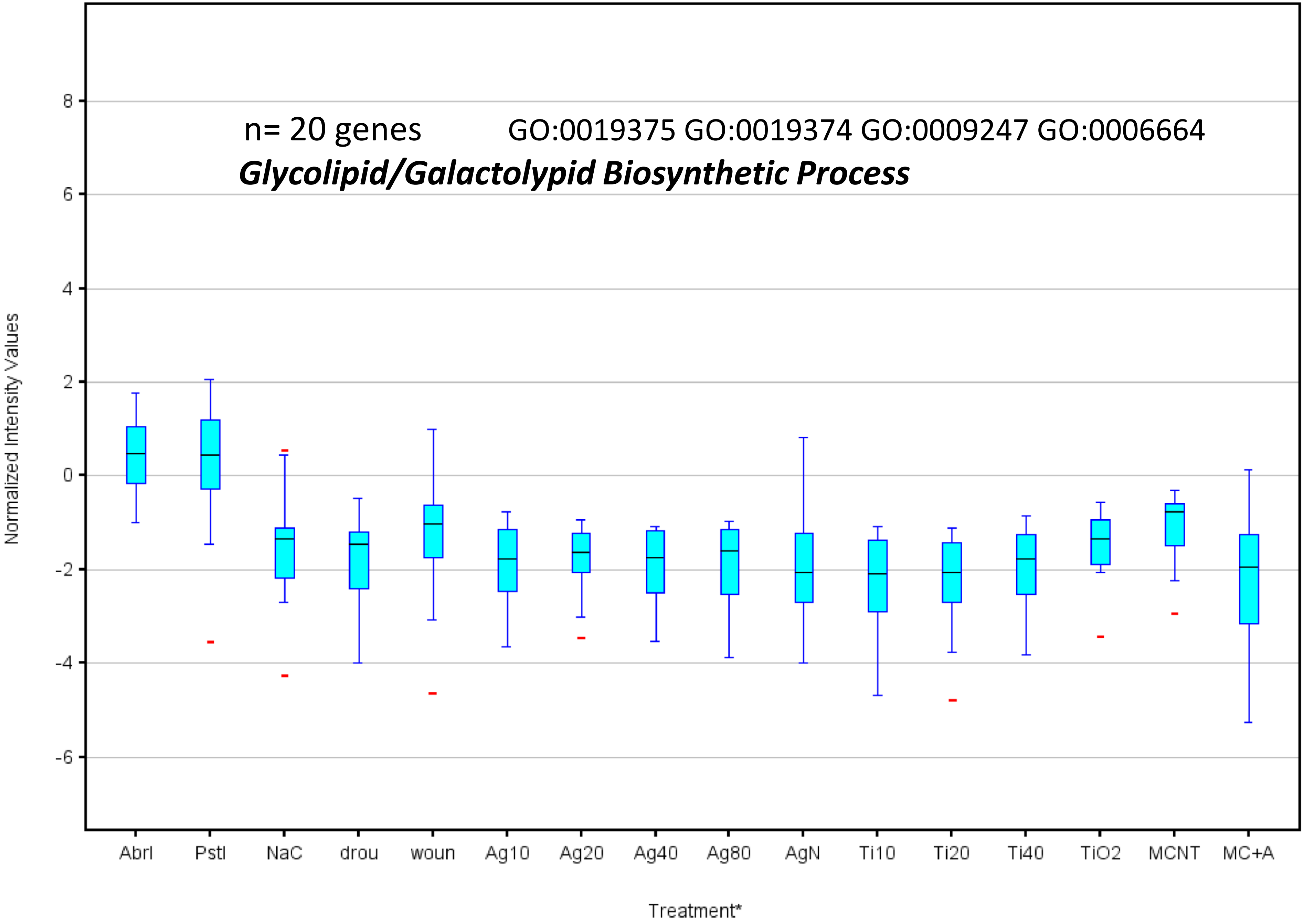
n= 20 genes
GO:0019375 GO:0019374 GO:0009247 GO:0006664
Glycolipid/Galactolypid Biosynthetic Process
Normalized Intensity Values
8
6
4
2
0
-2
-4
-6
Abrl
Pstl
NaC
drou
woun
Ag10
Ag20
Ag40
Ag80
AgN
Ti10
Ti20
Ti40
TiO2
MCNT
MC+A
Treatment*

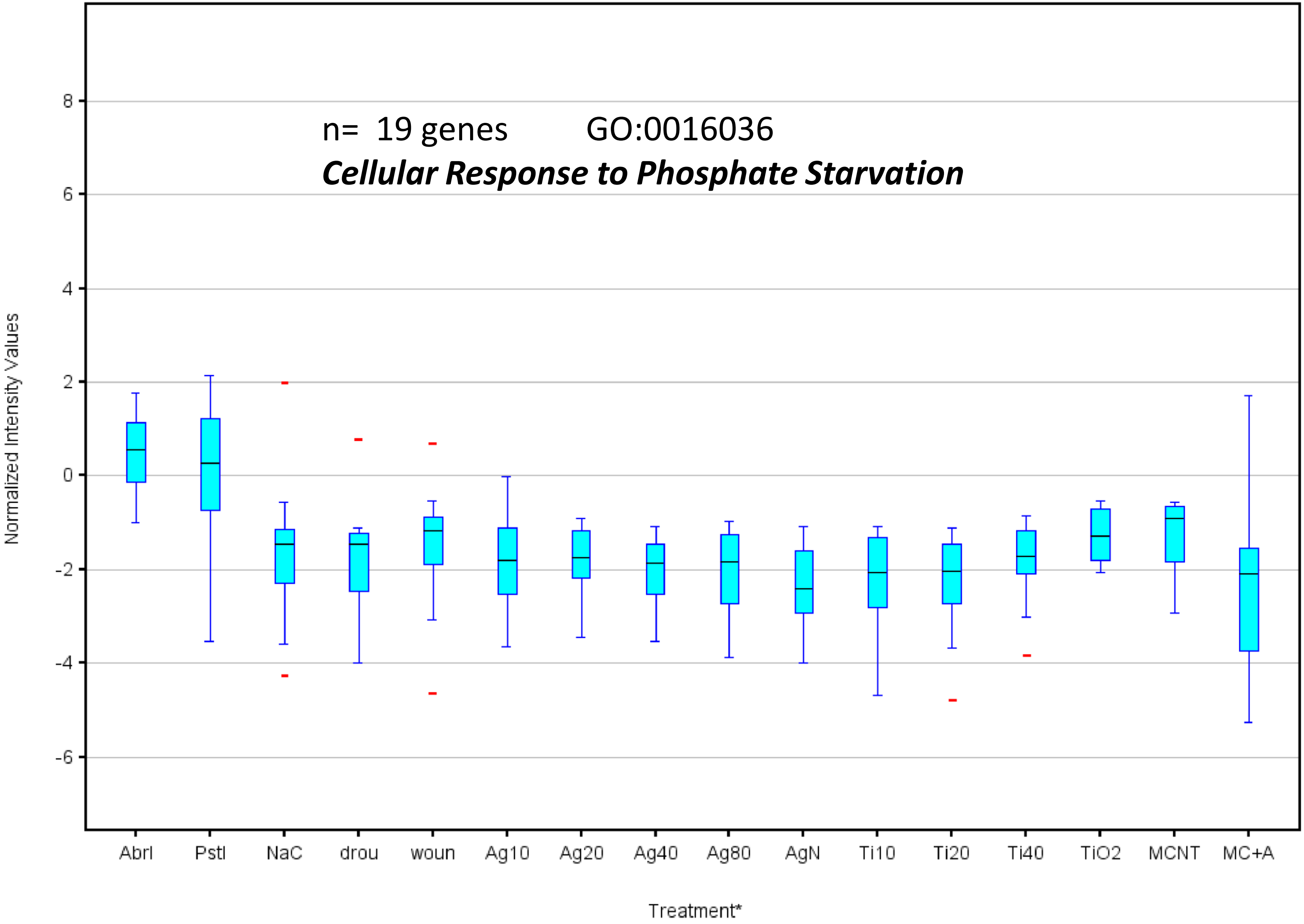
n= 19 genes GO:0016036
Cellular Response to Phosphate Starvation
Normalized Intensity Values
8
6
4
2
0
-2
-4
-6
Abrl
Pstl
NaC
drou
woun
Ag10
Ag20
Ag40
Ag80
AgN
Ti10
Ti20
Ti40
TiO2
MCNT
MC+A
Treatment*

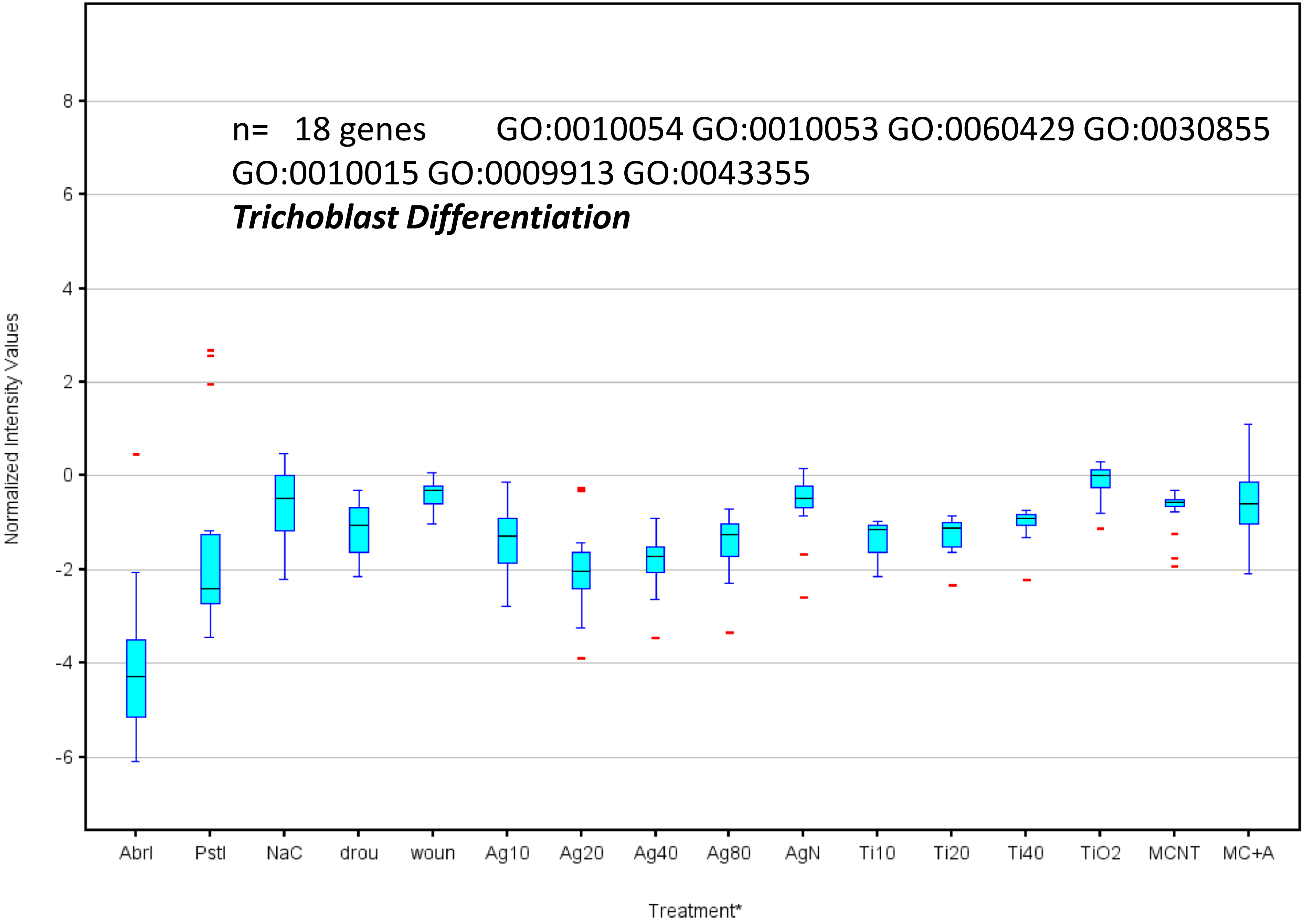

n= 18 genes GO:0010054 GO:0010053 GO:0060429 GO:0030855
GO:0010015 GO:0009913 GO:0043355
Trichoblast Differentiation
Normalized Intensity Values
8
6
4
2
0
-2
-4
-6
Abrl
Pstl
NaC
drou
woun
Ag10
Ag20
Ag40
Ag80
AgN
Ti10
Ti20
Ti40
TiO2
MCNT
MC+A
Treatment*

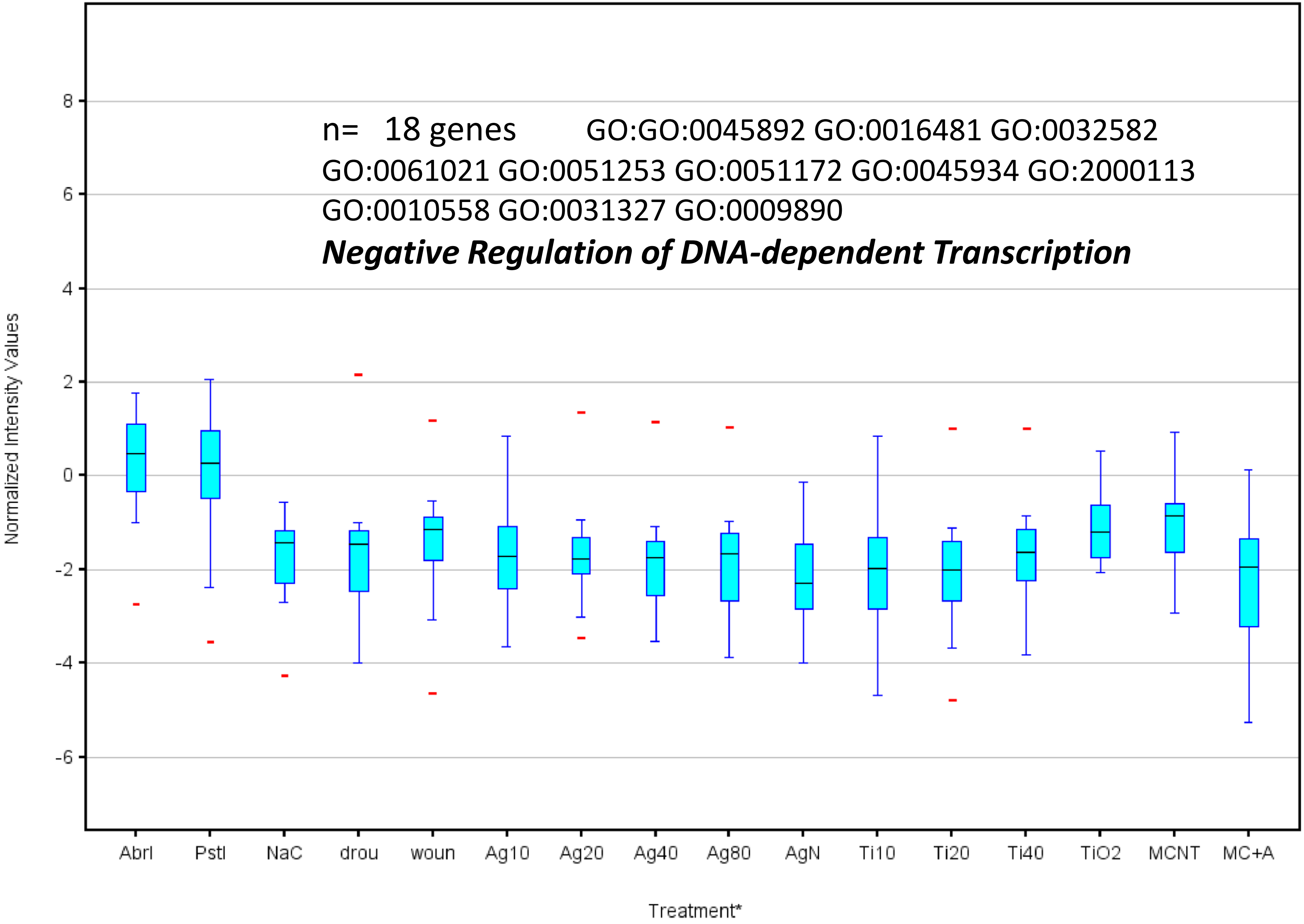
n= 18 genes GO:GO:0045892 GO:0016481 GO:0032582
GO:0061021 GO:0051253 GO:0051172 GO:0045934 GO:2000113
GO:0010558 GO:0031327 GO:0009890
Negative Regulation of DNA-dependent Transcription
Normalized Intensity Values
8
6
4
2
0
-2
-4
-6
Abrl
Pstl
NaC
drou
woun
Ag10
Ag20
Ag40
Ag80
AgN
Ti10
Ti20
Ti40
TiO2
MCNT
MC+A
Treatment*

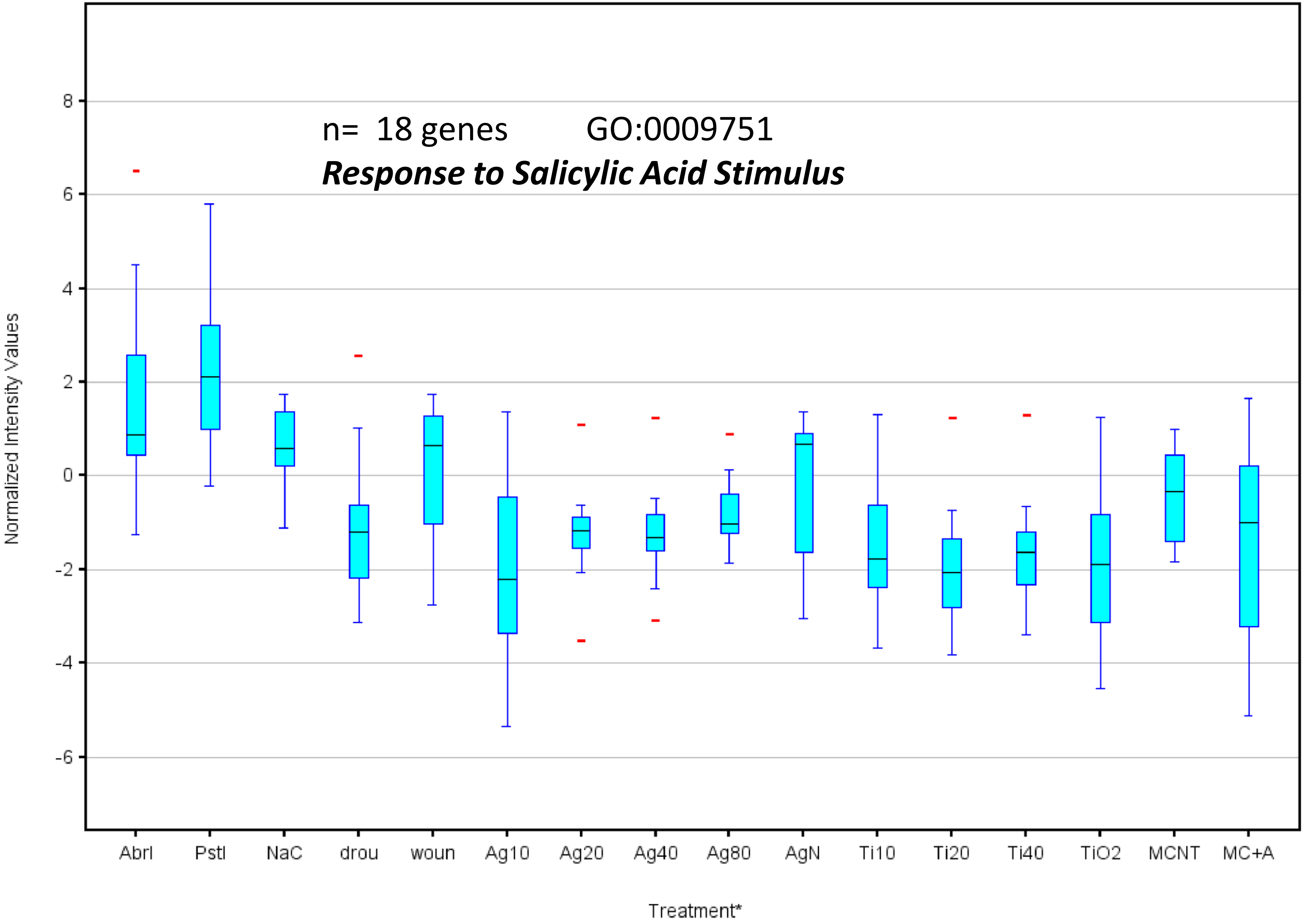
n= 18 genes GO:0009751
Response to Salicylic Acid Stimulus
Normalized Intensity Values
8
6
4
2
0
-2
-4
-6
Abrl
Pstl
NaC
drou
woun
Ag10
Ag20
Ag40
Ag80
AgN
Ti10
Ti20
Ti40
TiO2
MCNT
MC+A
Treatment*

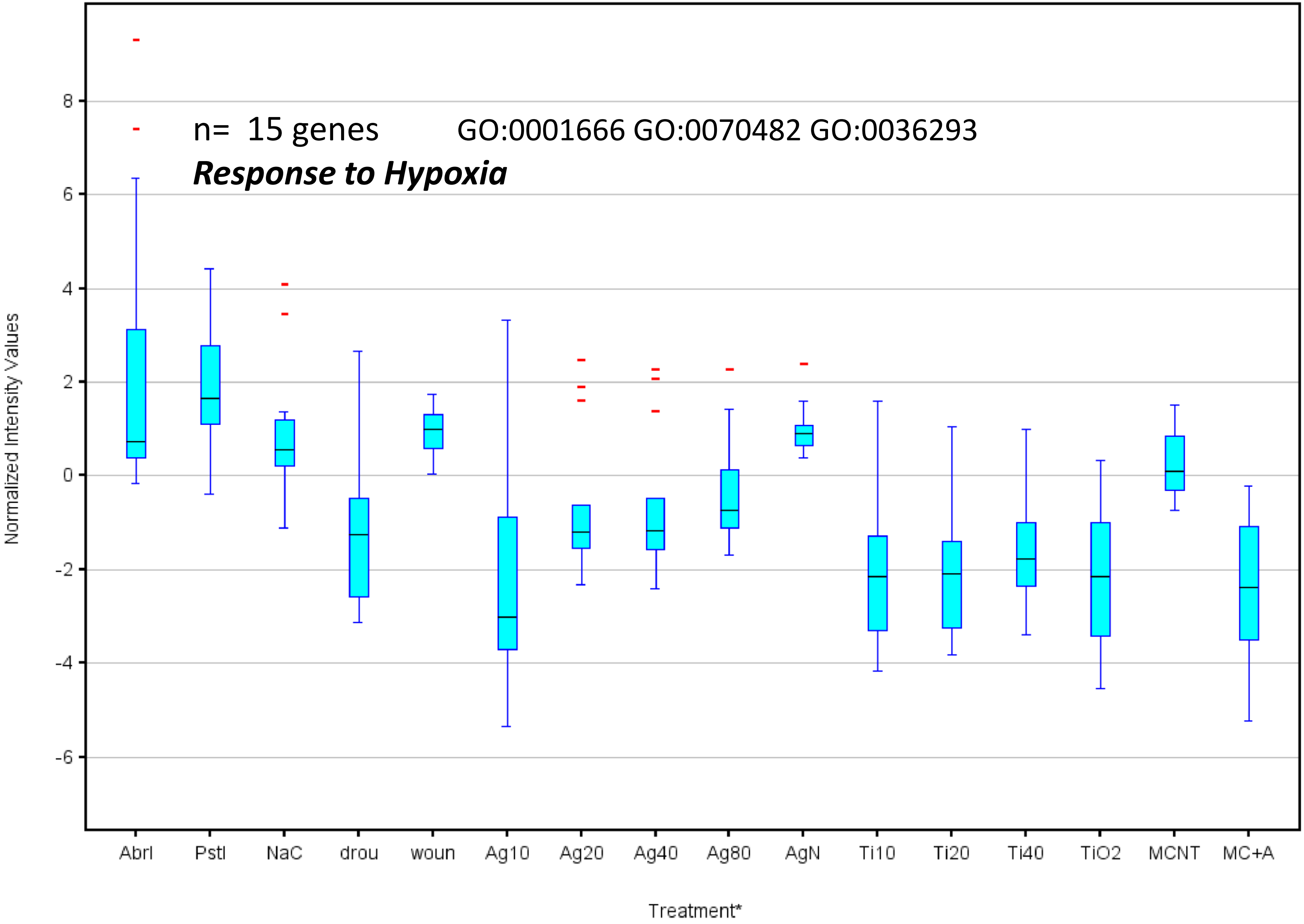
n= 15 genes
GO:0001666 GO:0070482 GO:0036293
Response to Hypoxia
Normalized Intensity Values
8
6
4
2
0
-2
-4
-6
Abrl
Pstl
NaC
drou
woun
Ag10
Ag20
Ag40
Ag80
AgN
Ti10
Ti20
Ti40
TiO2
MCNT
MC+A
Treatment*

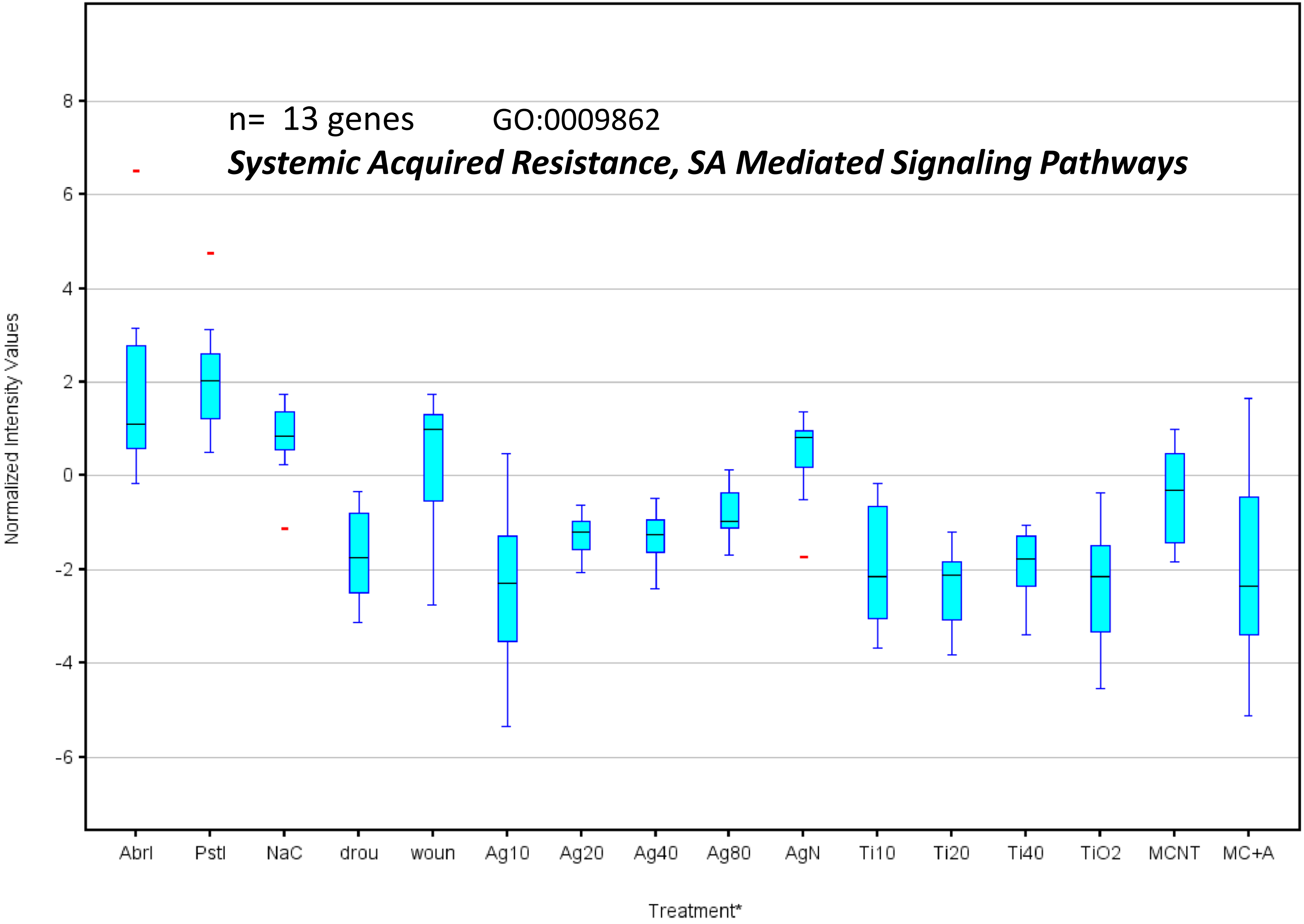
n= 13 genes
GO:0009862
Systemic Acquired Resistance, SA Mediated Signaling Pathways
Normalized Intensity Values
8
6
4
2
0
-2
-4
-6
Abrl
Pstl
NaC
drou
woun
Ag10
Ag20
Ag40
Ag80
AgN
Ti10
Ti20
Ti40
TiO2
MCNT
MC+A
Treatment*

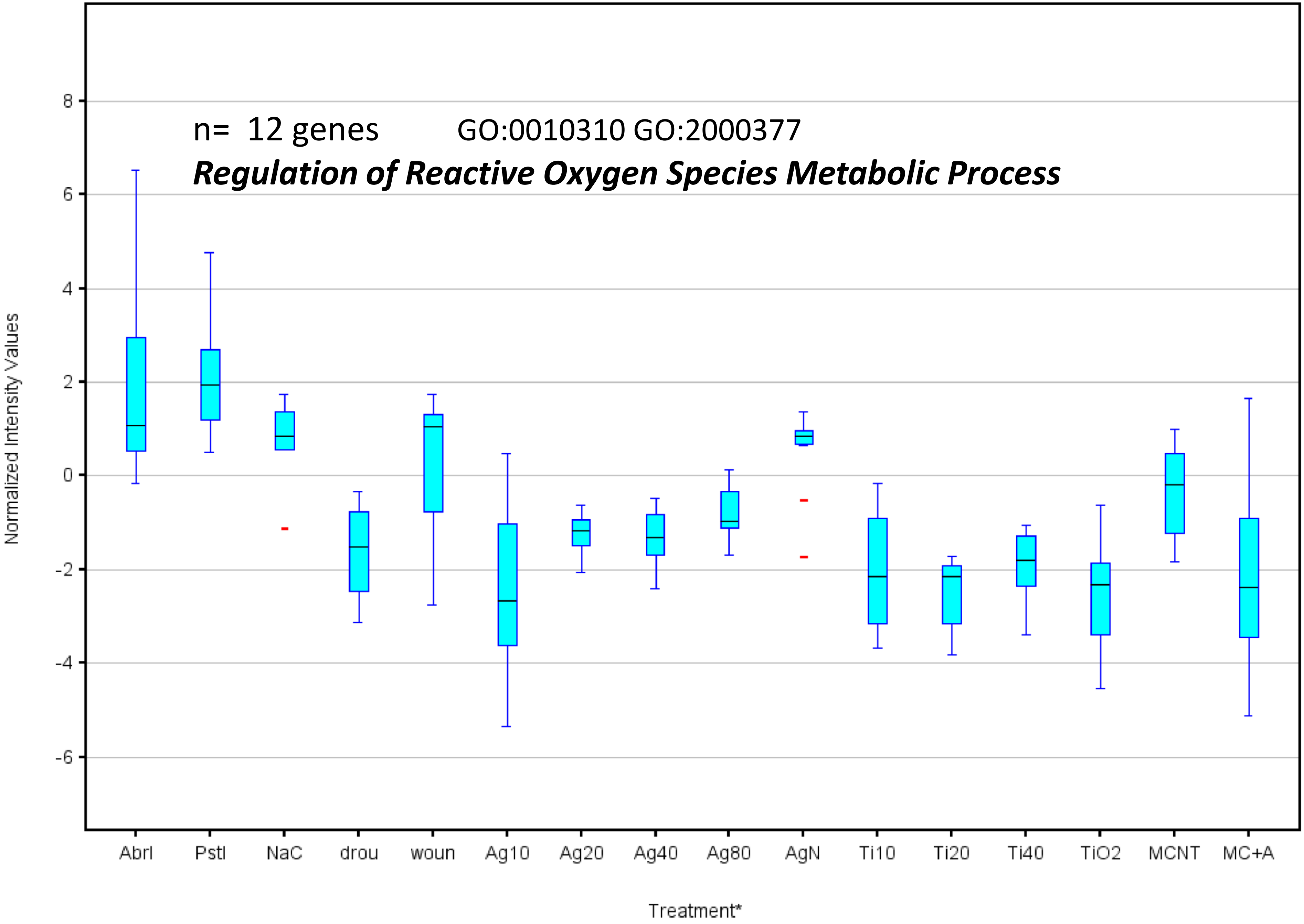
n= 12 genes GO:0010310 GO:2000377
Regulation of Reactive Oxygen Species Metabolic Process
Normalized Intensity Values
8
6
4
2
0
-2
-4
-6
Abrl
Pstl
NaC
drou
woun
Ag10
Ag20
Ag40
Ag80
AgN
Ti10
Ti20
Ti40
TiO2
MCNT
MC+A
Treatment*

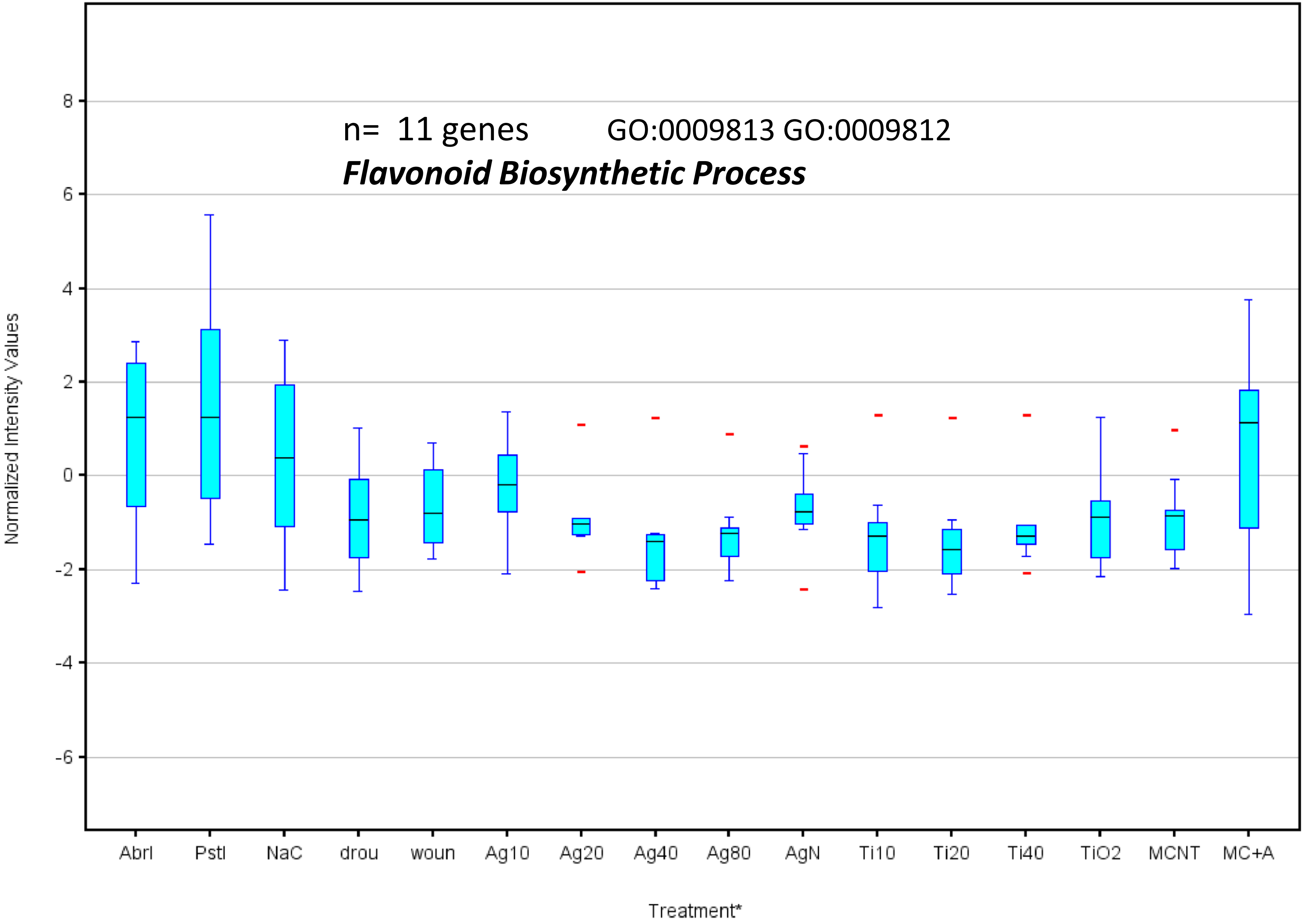
n= 11 genes GO:0009813 GO:0009812
Flavonoid Biosynthetic Process
Normalized Intensity Values
8
6
4
2
0
-2
-4
-6
Abrl
Pstl
NaC
drou
woun
Ag10
Ag20
Ag40
Ag80
AgN
Ti10
Ti20
Ti40
TiO2
MCNT
MC+A
Treatment*

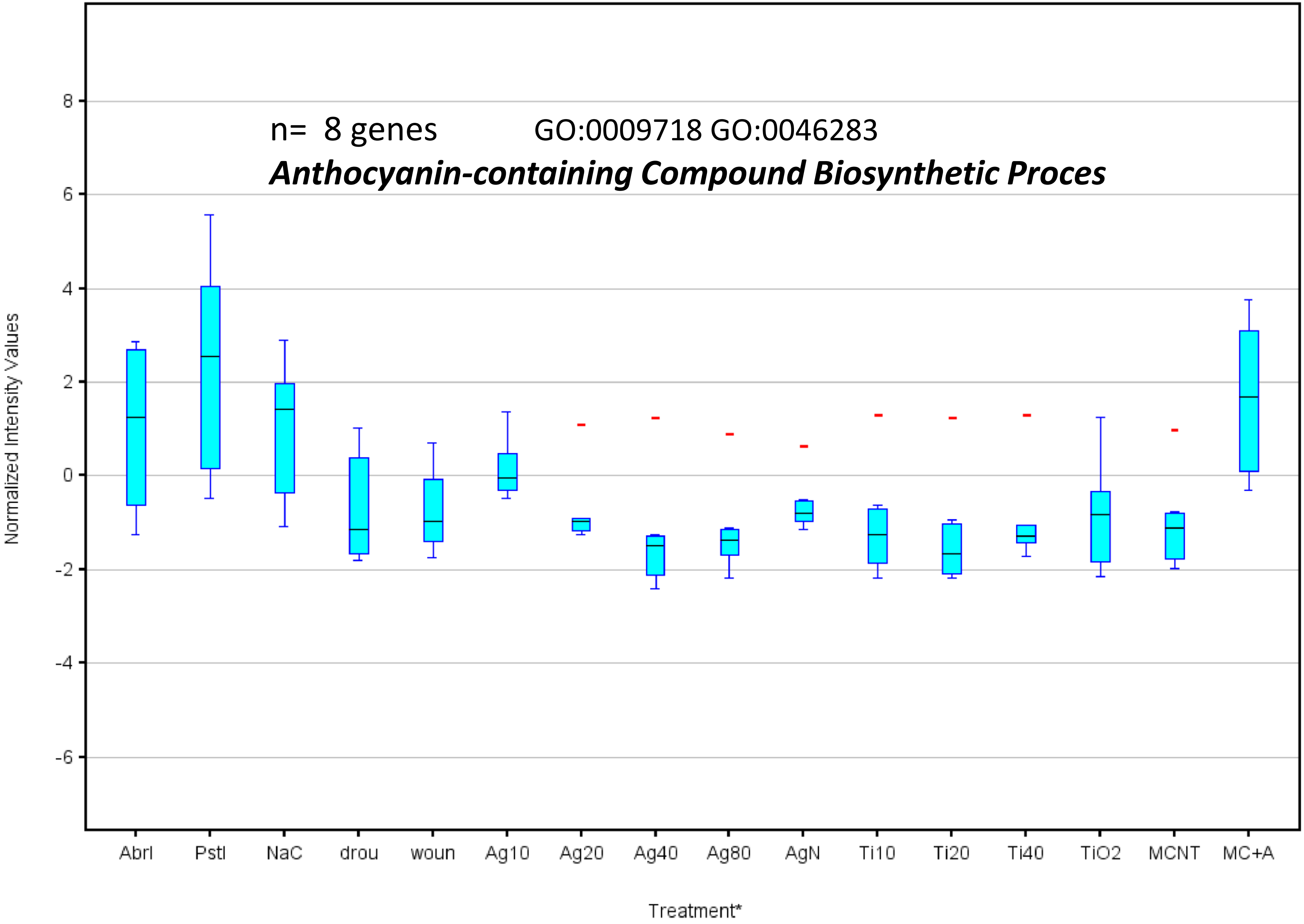
n= 8 genes GO:0009718 GO:0046283
Anthocyanin-containing Compound Biosynthetic Proces
Normalized Intensity Values
8
6
4
2
0
-2
-4
-6
Abrl
Pstl
NaC
drou
woun
Ag10
Ag20
Ag40
Ag80
AgN
Ti10
Ti20
Ti40
TiO2
MCNT
MC+A
Treatment*

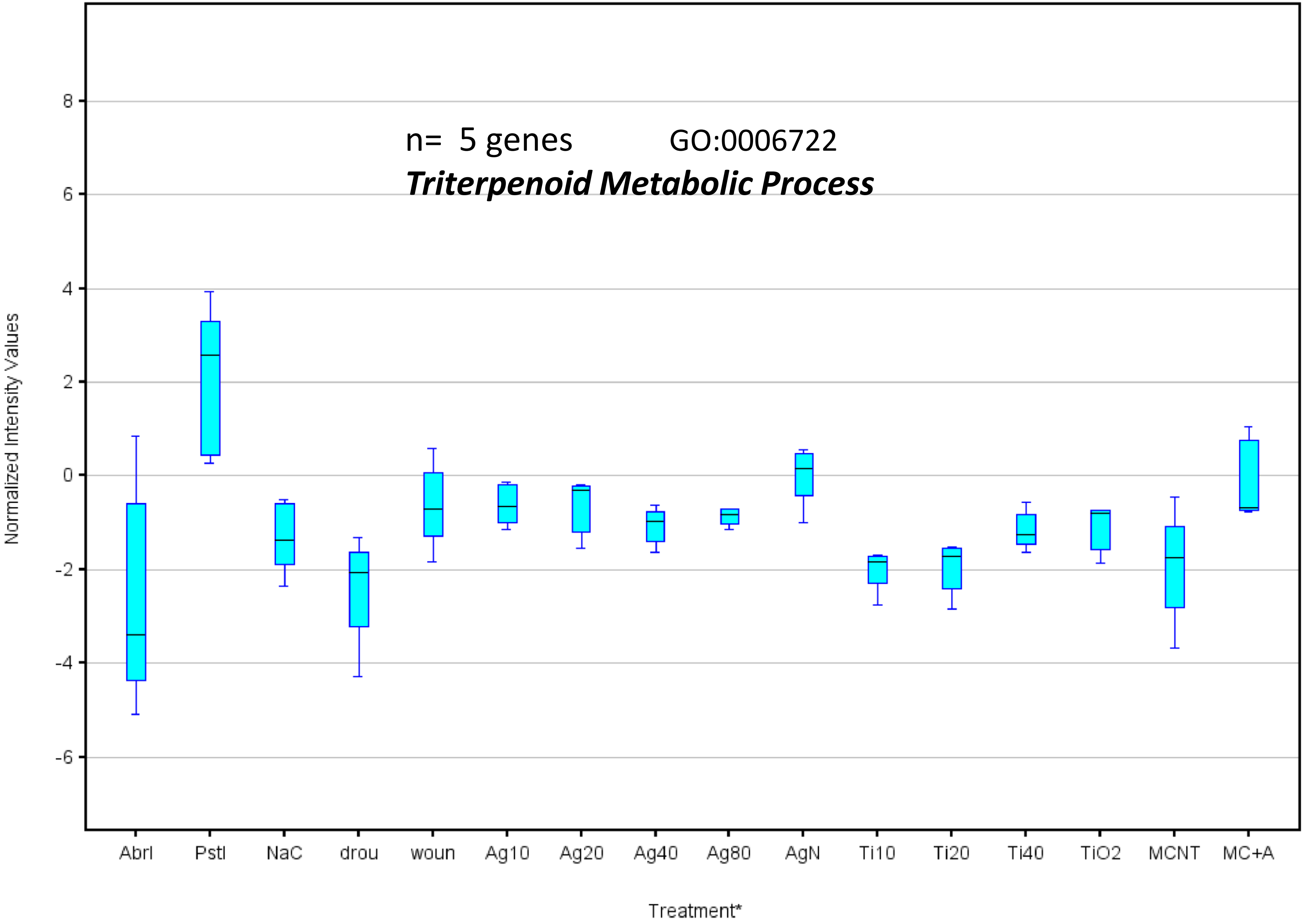

n= 5 genes
GO:0006722
Triterpenoid Metabolic Process
Normalized Intensity Values
8
6
4
2
0
-2
-4
-6
Abrl
Pstl
NaC
drou
woun
Ag10
Ag20
Ag40
Ag80
AgN
Ti10
Ti20
Ti40
TiO2
MCNT
MC+A
Treatment*

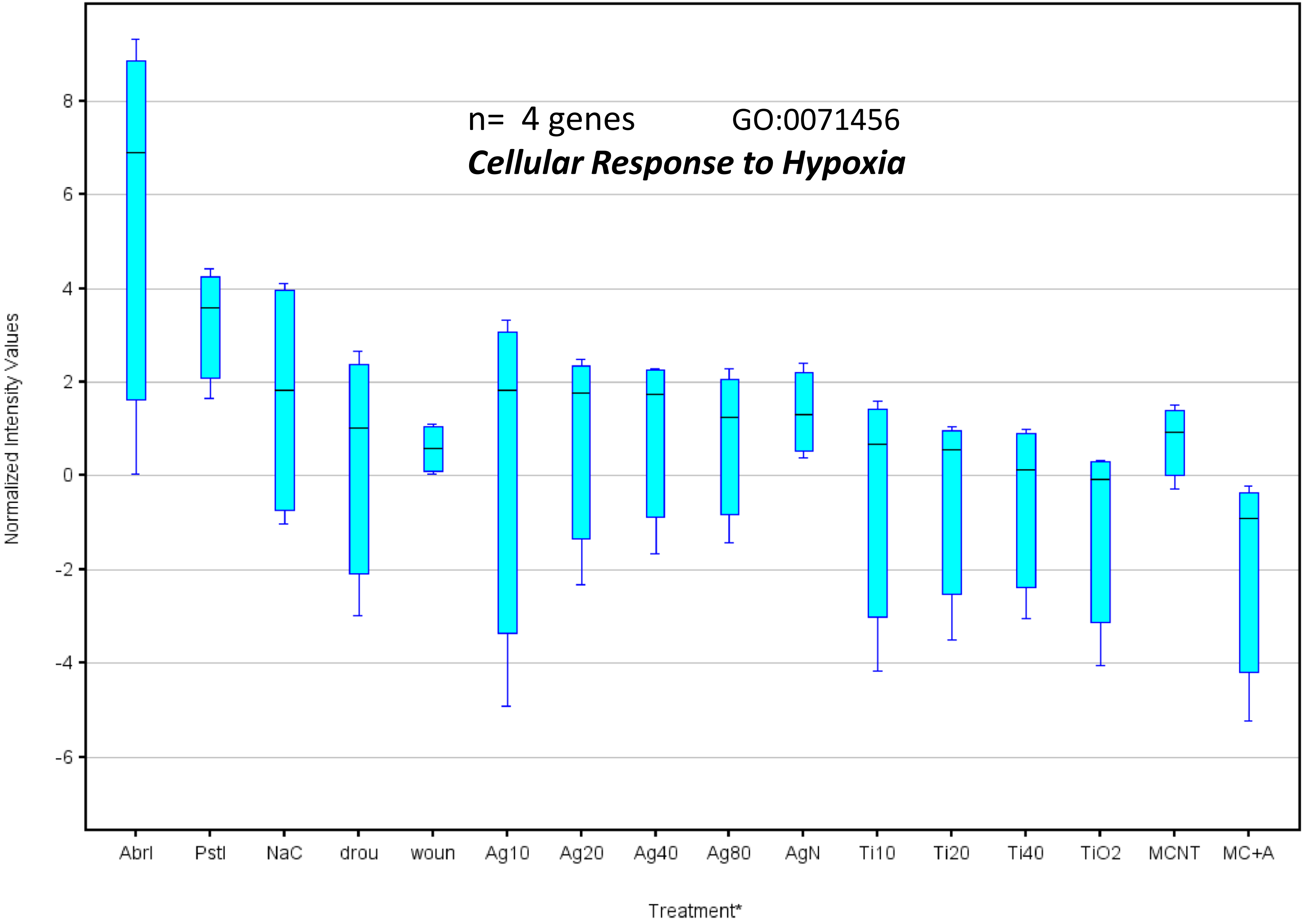

n= 4 genes GO:0071456
Cellular Response to Hypoxia
Normalized Intensity Values
8
6
4
2
0
-2
-4
-6
Abrl
Pstl
NaC
drou
woun
Ag10
Ag20
Ag40
Ag80
AgN
Ti10
Ti20
Ti40
TiO2
MCNT
MC+A
Treatment*
