# Supplementary material for: Early response to nanoparticles in the Arabidopsis transcriptome compromises plant defence and root-hair development through salicylic acid signalling
Source: BMC Genomics. 2015 Apr 24;16(1):341. doi: 10.1186/s12864-015-1530-4 (PMC4417227; doi:10.1186/s12864-015-1530-4)
Supplement: Additional file 9: — Figure showing the clustering and distribution of expression ratios of root-hair-cell and non-hair-cell genes. [file 12864_2015_1530_MOESM9_ESM.pdf]

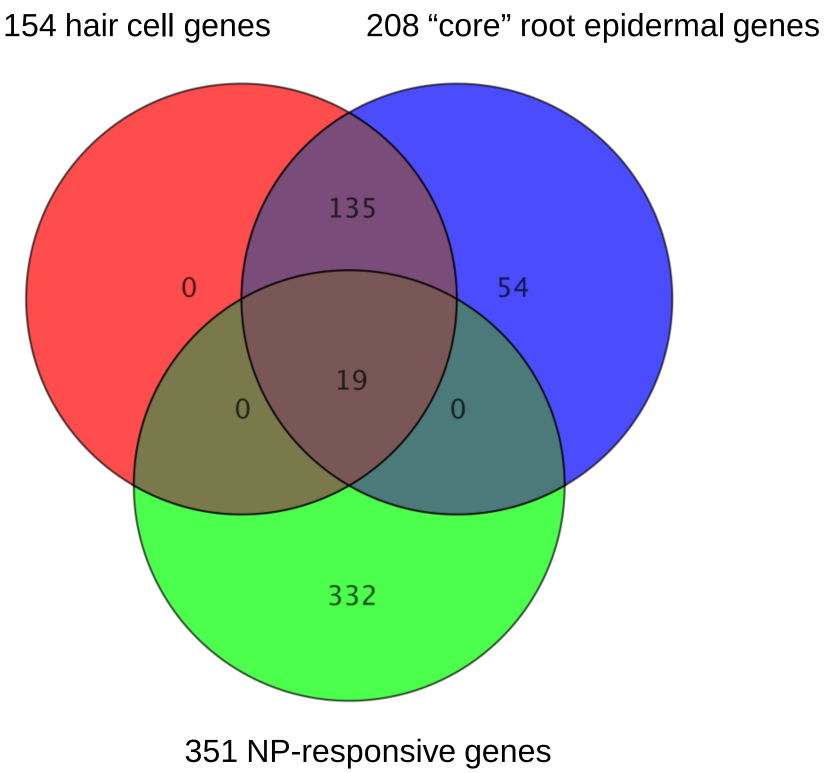

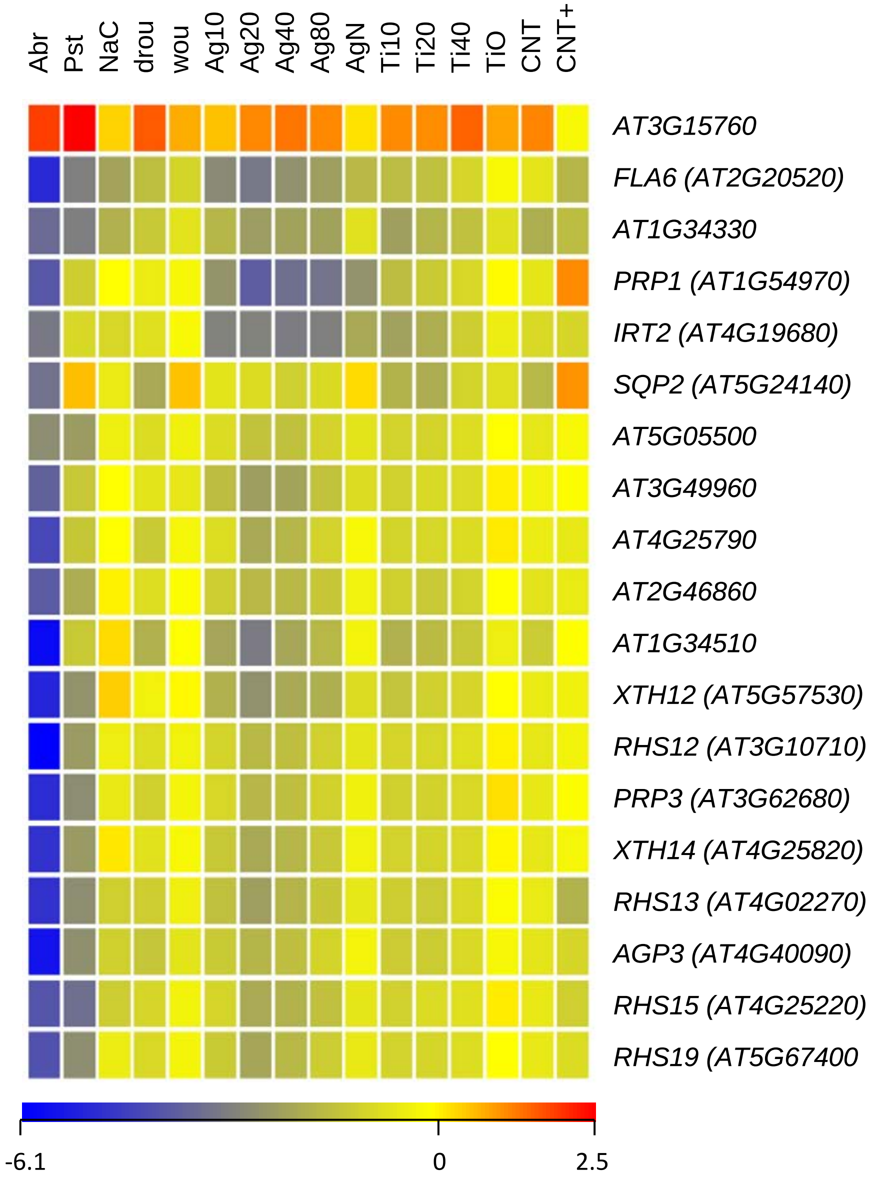

19 hair cell genes within the 351 gene-set

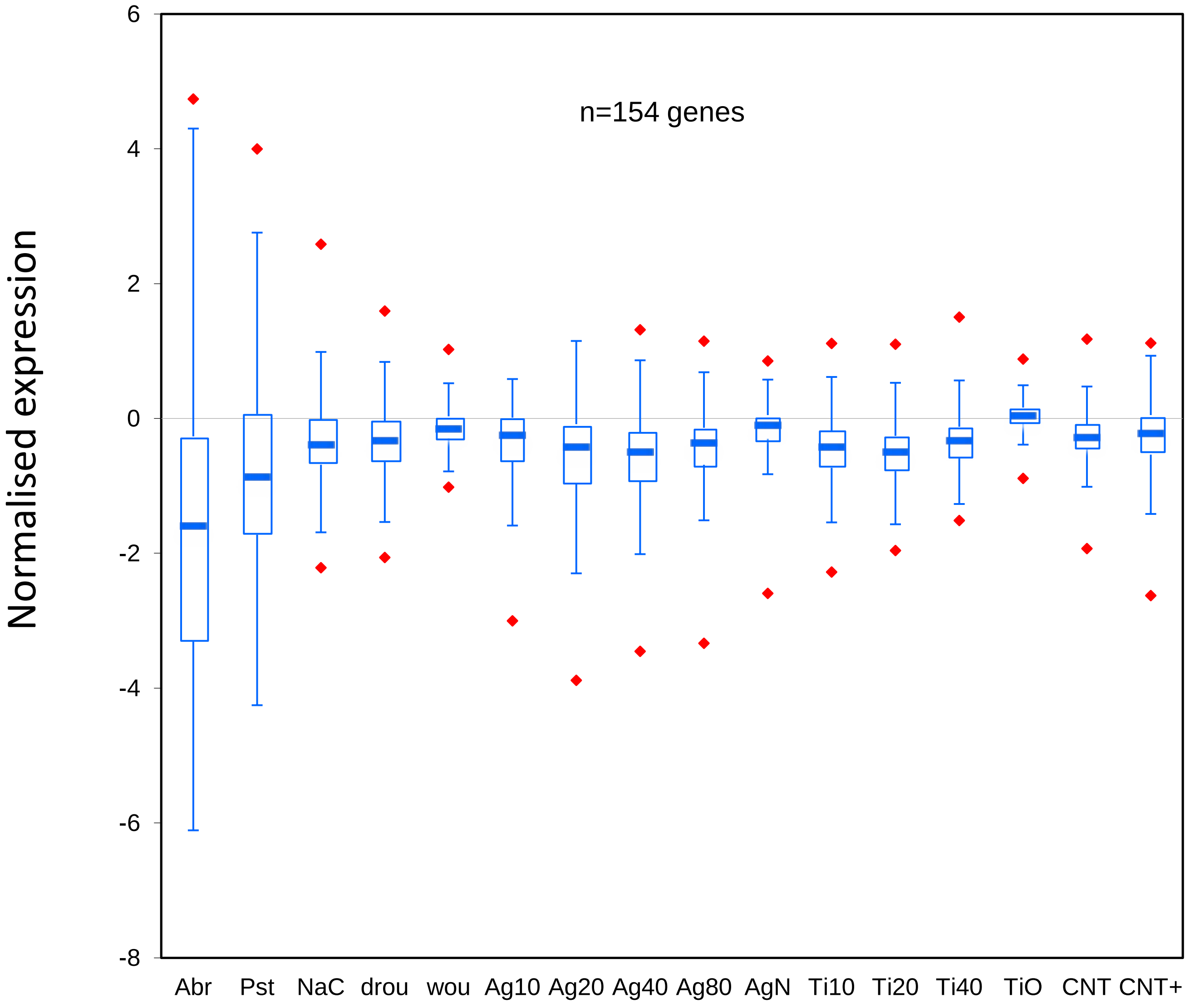

n=154 genes
Normalised expression
6
4
2
0
-2
-4
-6
-8
Abr
Pst
NaC
drou
wou
Ag10
Ag20
Ag40
Ag80
AgN
Ti10
Ti20
Ti40
TiO
CNT
CNT+

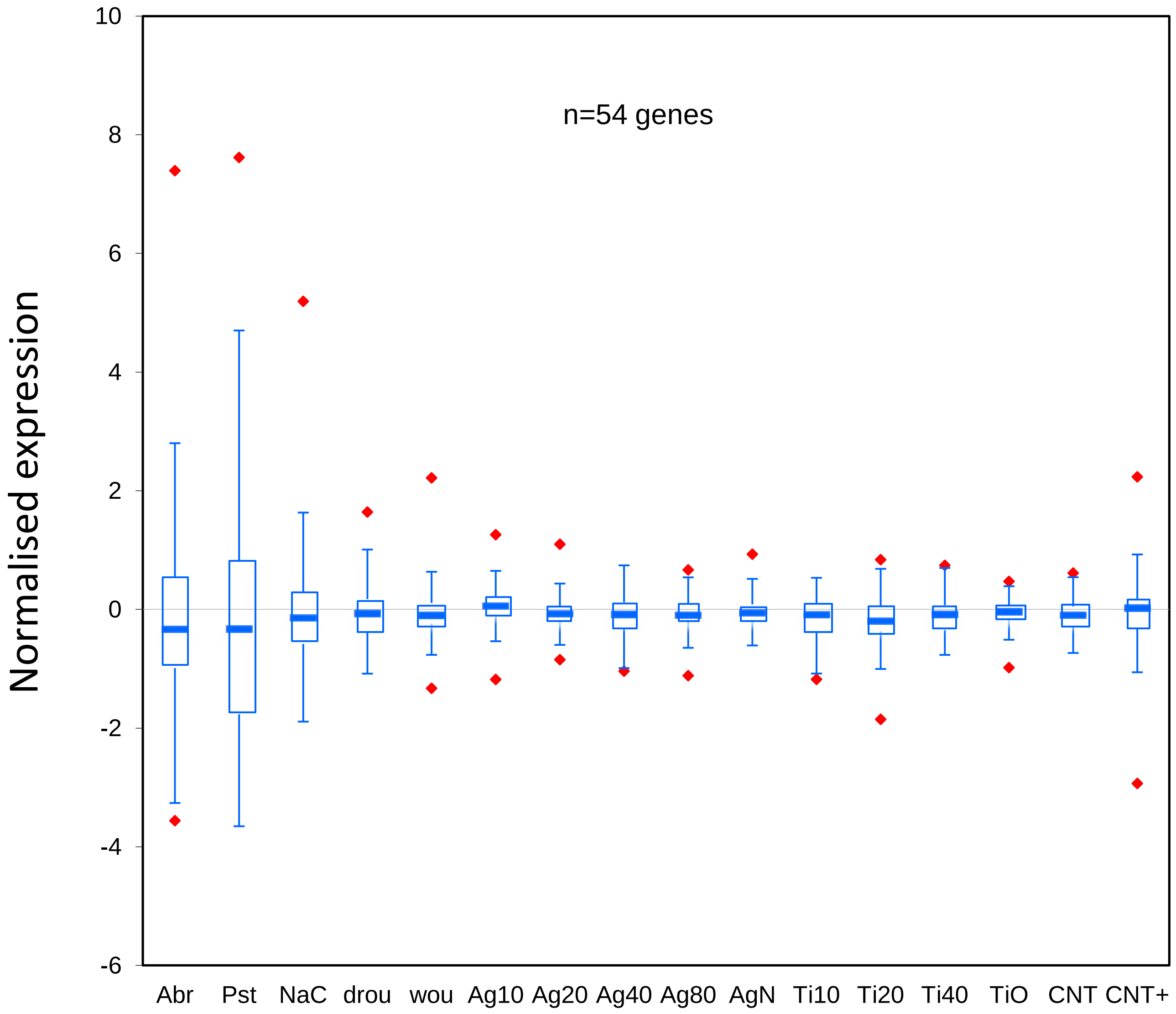

n=54 genes
Normalised expression
10
8
6
4
2
0
-2
-4
-6
Abr
Pst
NaC
drou
wou
Ag10
Ag20
Ag40
Ag80
AgN
Ti10
Ti20
Ti40
TiO
CNT
CNT+
