# Supplementary material for: Early response to nanoparticles in the Arabidopsis transcriptome compromises plant defence and root-hair development through salicylic acid signalling
Source: BMC Genomics. 2015 Apr 24;16(1):341. doi: 10.1186/s12864-015-1530-4 (PMC4417227; doi:10.1186/s12864-015-1530-4)
Supplement: Additional file 6: — Figure showing the clustering view of the genes involved in SAR. [file 12864_2015_1530_MOESM6_ESM.pdf]

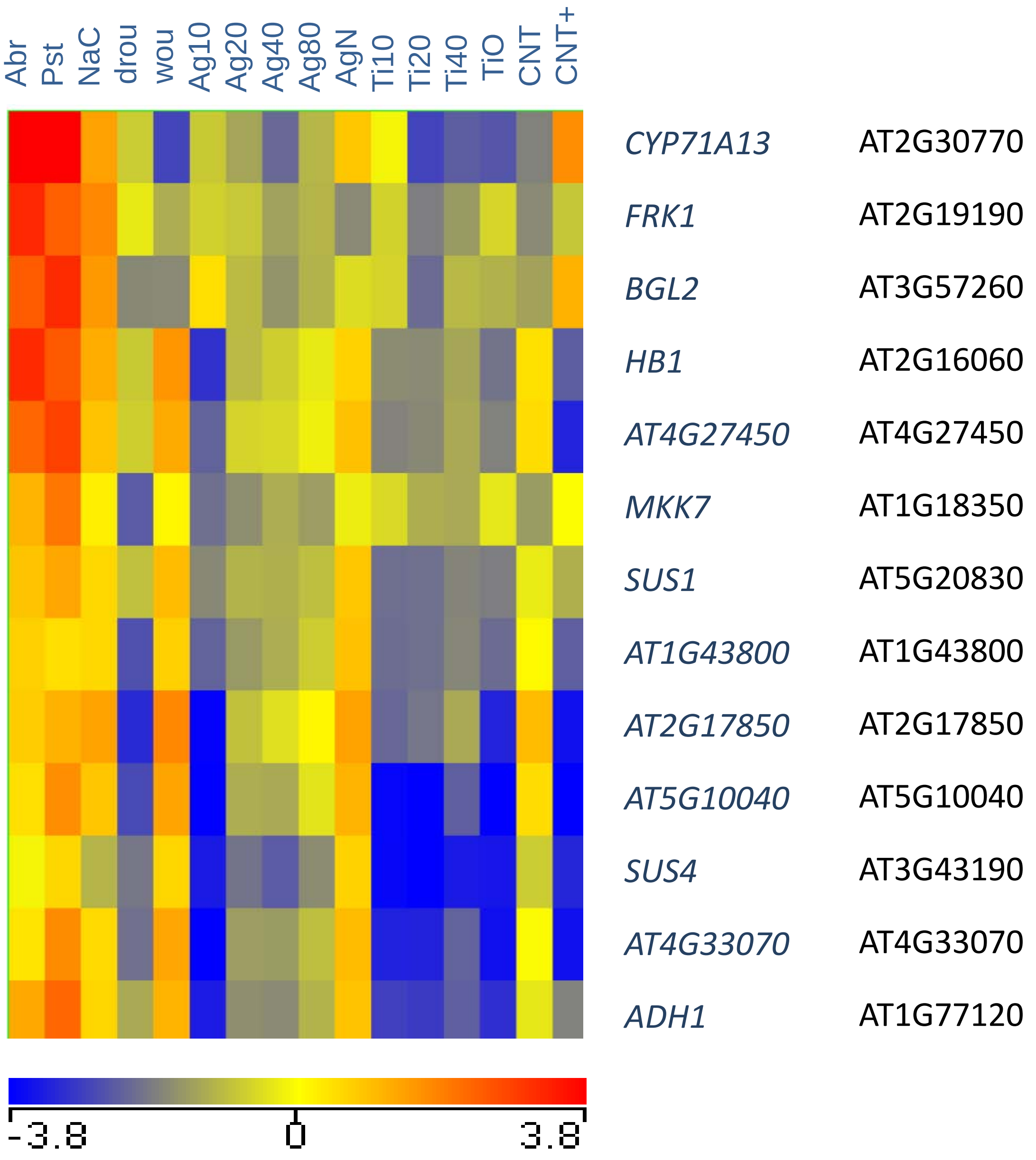
Abr
Pst
NaC
drou
wou
Ag10
Ag20
Ag40
Ag80
AgN
Ti10
Ti20
Ti40
TiO
CNT
CNT+
CYP71A13 AT2G30770
FRK1 AT2G19190
BGL2 AT3G57260
HB1 AT2G16060
AT4G27450 AT4G27450
MKK7 AT1G18350
SUS1 AT5G20830
AT1G43800 AT1G43800
AT2G17850 AT2G17850
AT5G10040 AT5G10040
SUS4 AT3G43190
AT4G33070 AT4G33070
ADH1 AT1G77120
-3.8
0
3.8
